# Intestinal microbiota influences clinical outcome and side effects of early breast cancer treatment

**DOI:** 10.1038/s41418-021-00784-1

**Published:** 2021-05-07

**Authors:** Safae Terrisse, Lisa Derosa, Valerio Iebba, François Ghiringhelli, Ines Vaz-Luis, Guido Kroemer, Marine Fidelle, Stergios Christodoulidis, Nicola Segata, Andrew Maltez Thomas, Anne-Laure Martin, Aude Sirven, Sibille Everhard, Fanny Aprahamian, Nitharsshini Nirmalathasan, Romy Aarnoutse, Marjolein Smidt, Janine Ziemons, Carlos Caldas, Sibylle Loibl, Carsten Denkert, Sylvere Durand, Claudia Iglesias, Filippo Pietrantonio, Bertrand Routy, Fabrice André, Edoardo Pasolli, Suzette Delaloge, Laurence Zitvogel

**Affiliations:** 1 Gustave Roussy Cancer Center, Villejuif, France; 2 INSERM U1015, Equipe Labellisée par la ligue Contre le Cancer, Villejuif, France; 3 University Paris Saclay, School of Medicine, Le Kremlin-Bicêtre, France; 4 Department of Medical Oncology, Saint Louis Hospital, Paris, France; 5 Center of Clinical Investigations in Biotherapies of Cancer (CICBT), Villejuif, France; 6 Research Platform in Biological Oncology, Dijon, France; 7 GIMI Genetic and Immunology Medical Institute, Dijon, France; 8 University of Burgundy-Franche Comté, Dijon, France; 9 Department of Medical Oncology, Center GF Leclerc, Dijon, France; 10 INSERM U 981, Gustave Roussy, Villejuif, Île-de-France France; 11 Department of Medicine, Breast Cancer committee, Gustave Roussy, Villejuif, France; 12 INSERM U1138, Equipe Labelisée par la ligue Contre le Cancer, Centre de Recherche des Cordeliers, Paris, France; 13 Cell Biology and Metabolomics Platforms, Gustave Roussy Cancer Campus, Villejuif, France; 14 Université de Paris, Paris, France; 15 Sorbonne Université, Paris, France; 16 University Paris Saclay, Saint-Aubain, France; 17 Prism Precision Medicine Center, Gustave Roussy, Villejuif, France; 18 Department of Medical Sciences, University of Trieste, Trieste, Italy; 19 Department CIBIO, University of Trento, Trento, Italy; 20 UNICANCER, Paris, France; 21 Faculty of Health, Medicine & Life Sciences, Department of Surgery, Maastricht, The Netherlands; 22 GROW School for Oncology & Developmental Biology, Maastricht, The Netherlands; 23 Maastricht University, Maastricht, The Netherlands; 24 Cancer Research UK Cambridge Institute, University of Cambridge, Li Ka Shing Centre, Robinson Way, Cambridge, UK; 25 Goethe University Frankfurt, Frankfurt, Germany; 26 Clinical Consultant Centre for Haematology and Oncology, Frankfurt, Germany; 27 Philipps-University Marburg and University Hospital Marburg (UKGM), Marburg, Germany; 28 Fondazione IRCCS Istituto Nazionale dei Tumori, Milano, Italy; 29 Division d’hémato-oncologie, Département de Médicine, Centre Hospitalier de l’université de Montréal (CHUM), Montréal, Québec Canada; 30 Department of Agricultural Sciences, University of Naples Federico II, Portici, Italy; 31 Task Force on Microbiome Studies, University of Naples Federico II, Naples, Italy

**Keywords:** Cancer, Translational research

## Abstract

The prognosis of early breast cancer (BC) relies on cell autonomous and immune parameters. The impact of the intestinal microbiome on clinical outcome has not yet been evaluated. Shotgun metagenomics was used to determine the composition of the fecal microbiota in 121 specimens from 76 early BC patients, 45 of whom were paired before and after chemotherapy. These patients were enrolled in the CANTO prospective study designed to record the side effects associated with the clinical management of BC. We analyzed associations between baseline or post-chemotherapy fecal microbiota and plasma metabolomics with BC prognosis, as well as with therapy-induced side effects. We examined the clinical relevance of these findings in immunocompetent mice colonized with BC patient microbiota that were subsequently challenged with histo-compatible mouse BC and chemotherapy. We conclude that specific gut commensals that are overabundant in BC patients compared with healthy individuals negatively impact BC prognosis, are modulated by chemotherapy, and may influence weight gain and neurological side effects of BC therapies. These findings obtained in adjuvant and neoadjuvant settings warrant prospective validation.

## Introduction

Breast cancer (BC) ranks first in women, and is the second cause of death in this gender. In addition to ageing and genetics, environmental factors (diet, ethanol consumption, endocrine disruptors, sedentary lifestyle) may contribute to the development of the disease. Several mechanisms might account for the putative impact of the microbiota on BC incidence or severity. Triple negative breast cancer (TNBC), whose cells do not express estrogen and progesterone hormone receptors and failed to express Her2 in in situ hybridization and immunohistochemistry, are kept in check by the immune system, as indicated by the predictive impact of tumor infiltrating lymphocytes on the efficacy of chemotherapy and prognosis [1–3] and the clinical benefit mediated by immune checkpoint inhibitors in PDL-1 expressing TNBC [4]. Accumulating evidence points to the capacity of the intestinal ecosystem, and more specifically specific health-associated bacteria commensals to dampen systemic inflammation [5, 6], to shape the innate and adaptive immune tonus [7–10], and reprogram the tumor microenvironment in tumor bearing mice [11, 12] and patients treated with immunotherapy [13, 14]. Retrospective and prospective studies conducted in lung, kidney, and melanoma cancers highlighted that antibiotics (ATB) negatively impact cancer patients’ clinical outcome during therapy with anti-PD1, or anti-PDL-1 antibodies [15–18]. In parallel, metagenomics analyses scrutinizing the repertoire of intestinal commensalism indicated that the alpha and/or beta diversity as well as the precise taxonomic composition of the microflora dictate the prognosis of patients with melanoma [12, 14], kidney [19], and lung [11] cancer. However, no data are currently available on the impact of the gut microbiota in shaping cancer immune surveillance in the context of TNBC.

Of note, on theoretical grounds, hormone receptor positive (HR^+^) BC may sense the gut microbiome in a different way than TNBC. Pioneering findings implicated that long-term estrogen supplementation (which is usually applicated for alleviation of menopausal symptoms) impacted composition of gut microbiota and microbial activity, and affected estrogen metabolism in the gut of mice [20]. The diversity and composition of the gut microbiota may increase BC risk by modulating systemic levels of estrogens and inflammation [21].

Pioneering reports also underscored the positive effects mediated by dietary interventions on BC incidence or prognosis [22]. Nicotinamide that can be produced by microbial components of the gut could reduce breast tumorigenesis in a T and NK cell-dependent manner [23]. In mice and patients with HR^+^ BC, periodic fasting or a fasting-mimicking diet enhanced the activity of the endocrine therapeutics by lowering circulating insulin, insulin-like growth factor-1, and leptin and by inhibiting trophic signaling by the AKT–mTOR pathway [24]. Soy isoflavone influenced the composition of the gut microbiota and tumor aggressiveness in immunodeficient mice humanized by fecal transfer of BC stools and inoculated with human BC implants [25].

Thus far, very few studies investigated the taxonomic diversity of commensals in women diagnosed with early BC. Hence, time is ripe to study the microbiota repertoire, its modulation by adjuvant treatments and its relevance for the outcome and side effects of therapies. Here we unveil for two independent cohorts of early BC that specific metagenomics species influence BC prognosis and neurological, endocrine and digestive side effects of chemo-hormono-therapy, opening new avenues for the future clinical management of BC.

## Results

### Fecal microbiota composition at diagnosis is associated with prognosis of early BC

We determined the metagenomics (MG)-based composition of stools at diagnosis of early BC in a subgroup (*N* = 76) of a deep cohort called “CANTO” (for CANcer TOxicities, NCT01993498). The CANTO study aims at performing a long-term follow-up of 13 250 women treated for BC over a period of ten years in order to quantify and prevent chronic toxicities related to treatment (surgery, radiation therapy, chemo-hormono-therapy) (refer to Consort diagram, Supplementary Fig. S1). The present analysis focuses on a first set of 9595 pts Enrolled from 2012–2017 with sufficient follow-up and mature data in 2019. Among the 76 females that signed the informed consent to get MG stool analysis, the median age was 52 (Supplementary Table S1). About 43% patients presented with a T2-T3 tumor size, and 51% with a Scarff Bloom& Richardson Grade 3 (Gr 3) adenocarcinoma (Supplementary Table S1). Up to 42 and 34% of tumors expressed HR or HER2, respectively, (24% were TNBC) and 46% presented with lymph node involvement (at least 1 N+) (Table S1). Overall, 14% presented with AJCC stage III. The adjuvant (or neoadjuvant) therapy consisted in anthracycline-taxane (93%), taxane- (7%) based chemotherapy (CT) and/or hormonotherapy (62%) (Supplementary Table S1).

**Fig. 1:**
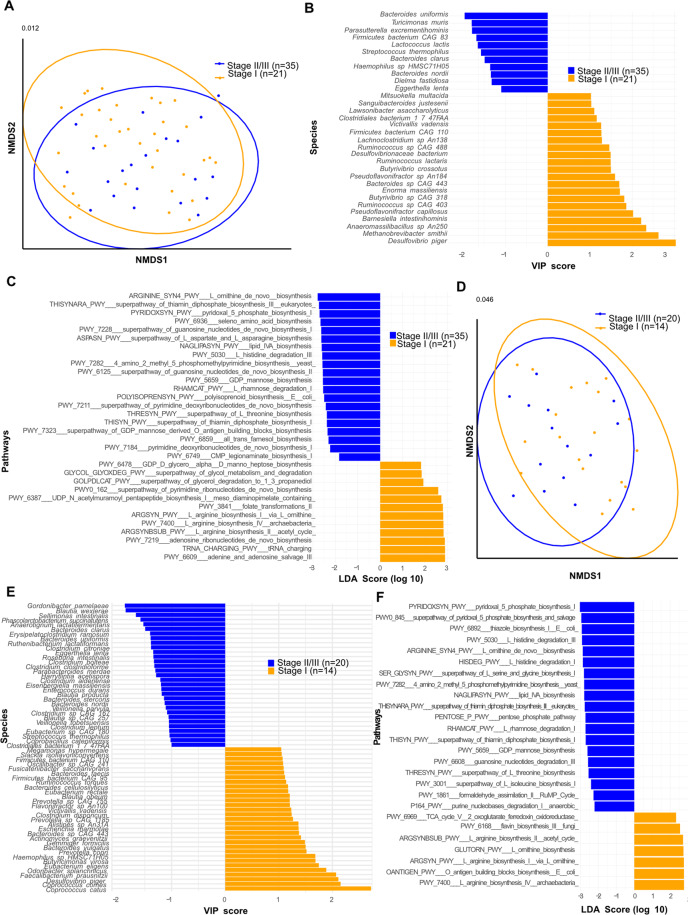
Metagenomics-based stool composition is associated with TNM staging in early breast cancer (BC) patients. **A** and **D**. Beta-diversity ordination plot based on principal coordinate analysis of normalized and standardized data of fecal microbiota composition before (**A**; pre-CT, A, *n* = 56) and after (**D**; post-CT, D, *n* = 34) adjuvant chemotherapy in patients with stage I (orange) and stage II-III (blue). The p-value is indicated at the top of the Y axis. **B** and **E**. Variable importance plot (VIP) scores were obtained within partial least square discriminant analysis by segregating stage I (orange) from stage II-III (blue), describing the most discriminant species in descending order of importance before (B; pre-CT-A, *n* = 56) and after E; post-CT, D, *n* = 34) adjuvant chemotherapy (only bacteria with prevalence >2.5% were taking into account). **C** and **F**. Differential abundances in terms of metabolic pathways between study groups (stage I vs stage II-III, pre- CT (**C**) vs post-**C**T (**F**)) by computing the effect size from linear discriminant analysis (LDA).

The composition of the gut microbiota ecosystem was evaluated using shot gun metagenomics (MG) profiling of early BC patients pre- (*N* = 76) and post-chemotherapy (CT) (*N* = 45), the post-chemotherapy specimen being paired with the pre-CT ones. To address whether the microbial composition differed between patients presenting with a favorable (T1, Gr 1, N-, AJCC stage 1) versus unfavorable BC prognosis (T2-3, Gr 3, N+, AJCC stage II/III), we performed univariate analysis of the variations in fecal microbial α diversity and provided principal coordinate analyses (PCoA) of microbial β-diversity distances within these patient subsets. Alpha diversity (observed OTU or Shannon index) did not significantly vary among these groups (not shown). Exploratory analysis of β-diversity (measuring sample diversity) was calculated using the Bray–Curtis measure of dissimilarity. As we observed that the fecal microbiota β-diversity discriminated groups of patients according to tumor size (< or >pT1, Supplementary Fig. S2A, C), to Scarff Bloom& Richardson grade (Gr1/2 *versus* Gr3, Supplementary Fig. S3A, B), to axillary node involvement (N- *versus* N+, Supplementary Fig. S4A, C) and to TNM staging (stage I *versus* stage II–III, Fig. 1A, D), we utilized a supervised analysis (Partial Least Squares Discriminant Analysis (PLS-DA) to explore the variance between the groups at baseline and post-CT (Fig. 1, Supplementary Figs. S2, S3, S4). To determine the relative contribution of the relative abundance of each bacterial species (spp) to the observed group separation at baseline and post-CT, bacterial spp were ordered according to their variable importance plot (VIP) score which relies on the supervised analysis of PLS-DA, comparing groups of patients according to each BC prognosis factor (< or >pT1 in Supplementary Fig. S2B, D, Gr1/2 versus Gr3 in Supplementary Fig. S3C, N- versus N+ in Supplementary Fig. S4B, D, and stage I versus stage II/III in Fig. 1B, E).

Several consortia of commensals were found iteratively overrepresented in patient subsets bearing more aggressive tumors (associated with larger tumors at diagnosis, axillary node involvement (N+), and UICC staging ≥II) in fecal specimen harvested pre- and/or post-CT, such as species belonging to the *Streptococcus* genera (*S. mitis, S. vestibularis*), Lachnospiraceae family members (*Sellimonas intestinalis*, *Eisenbergiella massiliensis, Blautia wexlerae)*, *Veillonella* genus (*V. atypica, V. parvula*), Bacteroides spp (P*. merdae, B. uniformis, B.xylanisolvens*…), *E. ramosum*, Enterobacteriaceae family members such as *Bilophila wadsworthia*, *Hafnia alvei and Klesiella spp*, Clostridiaceae family members *(C. spiroforme, C. asparagiforme, C. boltae)* that we previously reported in advanced NSCLC and kidney cancer patients manifesting a primary resistance to immune checkpoint inhibitors [11, 19] (Fig. 1, Supplementary Figs. S2B, D and S4B, D). Conversely, health-associated commensals, i.e., *Methanobrevibacter smithii* archae, Eubacteriaceae family members *(such as E. eligens, E. hallii, E. rectale), A. muciniphila, Defulfovibrio piger*, species belonging to the *Coprococcus* genus *(C. comes, C. catus)* and *Collinsella* genus *(C. aerofaciens), B. vulgatus* as well as Ruminococcaceae family members *(R. bicirculans, R. lactaris)* were reproducibly associated with N- or ≤pT1 BC in longitudinal samples (Fig. 1, Supplementary Figs. S2, S3, S4B–D). Of note, *A. muciniphila, C. aerofaciens,* and distinct *Bacteroides spp*. have already been linked to favorable outcome in advanced melanoma, NSCLC and kidney cancer patients responding to immune checkpoint inhibitors [10–12, 19]. The functional pathways associated with these bacterial patterns were different between good and bad prognosis BC, but similar pre-and post-CT (Fig. 1C, F). Increased biosynthesis of L-Arginine and adenosine ribonucleotides, dominance of oxoglutarate ferredoxin oxidoreduction, and adenine–adenosine salvage were observed in favorable BC, while biosynthesis of lipids, thiamine diphosphate, pyridoxal −5 phosphate, L-threonine and degradation of L -histidine were associated with poor-prognosis cancers (Fig. 1C, F).

**Fig. 2:**
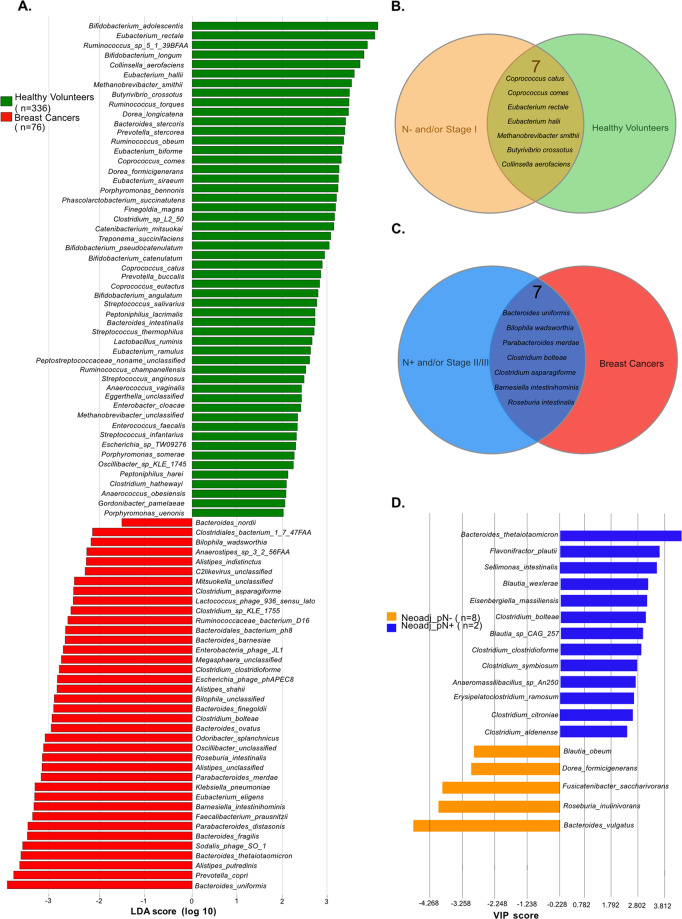
Metagenomics analyses of stool samples from BC patients compared with healthy volunteers (HV). **A** LEfSe (Linear discriminant analysis of effect size) method was used to detect differential abundant species (only bacteria with a prevalence >2.5% were considered) between (green bars, *n* = 336) and BC patients (BC, red bars, *n* = 76). Venn diagram describing the listing and the numbers of bacterial species in common between no pathological lymph node involvement (pN−) and HV (*n* = 7, **B**) and in common between pathological lymph node involvement (N+) and BC (*n* = 7, **C**). **D**. VIP scores by comparing species abundance according to the pathological lymph node involvement (no pathological lymph node involvement, pN-, orange; pathological lymph node involvement, pN+, blue) in stools collected after neoadjuvant CT (*n* = 10).

Next, to further analyze the clinical relevance of these commensals associated with favorable or unfavorable prognosis, we scrutinized fecal compositional differences between these 76 BC and 54 Italian healthy volunteers (HV), whose feces were harvested according to the same procedures and analyzed together with 282 HV-derived samples selected from referenced public metagenomes. Of note, these 282 HV were randomly permutated several times and analyzed for data validation (as detailed in M&M). Species with differential abundance between cancer free (HV) and BC groups were used as input for the linear discriminant analysis (LDA) to calculate an effect size (LEfSe method, LDA score > 2) (Fig. 2A). We found bacterial commonalities between N- groups and cancer free individuals (Fig. 2B) and between N+ and BC status (Fig. 2C). Hence, we identified 7 MG species associated with both a cancer -free status and with a more favorable prognosis, in case of cancer (mostly N- and/or TNM staging I) in post-CT specimen, such as *C. comes* and *C. catus, Collinsella aerofaciens, E. rectale*, *M. smithii*, or MG recovered in both pre- and post- CT feces (such as *B. crossotus, C. aerofaciens*) (Fig. 2B). Conversely, we identified 7 commensals characterizing BC patients and associated with worse prognosis in pre- or post-CT specimens such as *B. uniformis* and *C. bolteae* (N+ and TNM staging >I), *P. merdae, C. asparagiforme, R. intestinalis, B*. intestinihominis (Fig. 2C).

Next, we attempted to validate these findings in a second independent cohort of BC patients treated with neo-adjuvant CT enrolled in CANTO (*n* = 17 pre-CT, 10 post-CT) (Supplementary Table S1). Here again, the LEfSe method allowed to segregate baseline stool compositions of BC patients with post-chemotherapy pN+ versus pN- status (Fig. 2D) as well as pathological complete responders (stage 0) from non-complete responders (stage I–II) (Supplementary Fig. S3D). *B. vulgatus*, *Blautia obeum* and *Dorea formicigenerans* that were found overrepresented post-CT in stage I BC (Fig. 1E, Fig. 2A) stood out among the few taxons associated with the pN- status (metastasis-free axillary lymph nodes post-neoadjuvant CT) (Fig. 2D). In contrast, 8 MG species described in N+ or stage II-III post-CT (i.e., *E. ramosum*, *C. bolteae*, *C. citroniae*, *C. aldenense*, *C. clostridioforme*, *Blautia wexlerae*, *Sellimonas intestinalis*, *Eisenberghiella massiliensis*) and overabundant in BC patients as compared to HV (Fig. 2A) were also significantly associated with the pN+ status in post-neoadjuvant CT specimen, but not with cancer immunohistochemical subtype (Fig. 2D, Supplementary Fig. S3D).

Altogether, using four complementary methodologies of comparisons, sampling pre-CT, sampling post-CT, cancer -free versus cancer bearing status, and adjuvant *versus* neoadjuvant settings, we conclude that the intestinal ecosystem represents another variable to consider in women at early BC diagnosis, that possibly influences prognosis (as based on correlations with tumor size, histological grade, axillary LN involvement and staging). Of note, in this cohort of 76 early BC, MG stool composition was associated with the presence of axillary metastases in univariate analyses identically to the classical clinical prognostic parameters (Supplementary Table S2).

### Adjuvant chemotherapy modified the β diversity of fecal composition in early BC

**Fig. 3:**
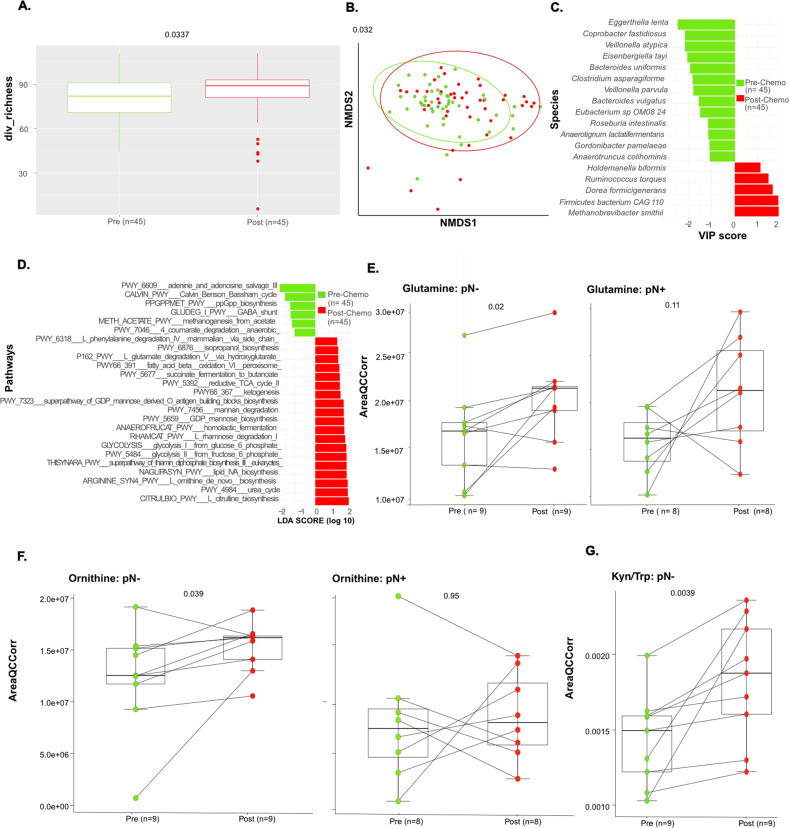
Chemotherapy significantly affected fecal composition in a paired sub-group analysis. **A**. Alpha diversity in terms of richness in longitudinal and paired stool samples (*n* = 45) collected before (pre-CT, green) and after (post-CT, red) CT. **B**. Beta-diversity ordination plot based on principal coordinate analysis of normalized and standardized data of fecal microbiota composition in pre-CT (green) and post-CT (red) stools. Most discriminative species (**C**; through VIP (implemented within partial least square discriminant analysis) and pathways (**D**; through LEfSe) by differentiating between pre-CT and post-CT samples stools. lasma levels of metabolites through high dimensional metabolomics according to pathological lymph node involvement (no pathological lymph node involvement, pN-; pathological lymph node involvement, pN+) in terms of Glutamine (in pN- patients, *n* = 18, left panels; and pN+ patients, *n* = 16, right panels). **E** Ornithine (in pN- patients, *n* = 18, left panels; and pN+ patients, *n* = 16, right panels) **F** and Kynurenine to Tryptophan (Kyn/Trp) ratio in pN- patients monitored in patients pre-CT, (green) and post-CT, (red), *n* = 18 (**G**). *P* values are indicated at the top of the *Y* axis (Wilcoxon test).

To characterize changes (if any) in microbial composition over the course of 8 cycles of CT, we first analyzed the variations in fecal microbial α diversity (Fig. 3A) and performed PCoA of microbial β diversity distances (Fig. 3B). Compared with baseline (prior to CT), α diversity (richness index) increased after CT (Fig. 3A, *p* = 0.033). When considering β diversity variations in a paired manner, we identified significant shifts in the microbial composition over time (Fig. 3B, *p* = 0.032). Chemotherapy increased the relative abundance of health-related MG species (such as *Methanobrevibacter smithii* archae associated with HV and pN- status in BC (Fig. 2B), *D. formicigenerans* (overabundant in HV, Fig. 2A), *R. torques* (associated with stage I BC, Fig. 1B, E pre-et post-CT, Fig. 3C). In contrast, CT tended to reduce the over-representation of bacteria associated with BC diagnosis and unfavorable prognosis (such as *C. asparagiforme, B. uniformis, Eggerthella lenta*, species from the *Veillonella* genus*)* (Fig. 3C). Of note, chemotherapy did not significantly affect the relative abundance and prevalence of the immunogenic species *A. muciniphila*, associated with ≤pT1, in pre-CT stool samples (not shown). Interestingly, the functional pathways associated with bacterial gene patterns were influenced by CT, which increased L-ornithine biosynthesis, glycolytic intermediates (glucose-6-phosphate and fructose-6-phosphate), L-glutamate degradation, lipid biosynthesis, and ketogenesis (Fig. 3D).

To validate these metabolic patterns associated with fecal content, we performed a metabolomic analysis of the plasma in 29 BC females (26 pre- and 29 post-CT samples) compared with age matched HV. Indeed, we confirmed that glutamine and L-ornithine plasma levels increased during CT, mostly in pN- (Fig. 3E, F). Conversely, the tryptophane/kynurenine degradation pathway, known to be involved in lymphocyte exhaustion and associated with advanced staging in BC stools (Supplementary Fig. S5A) was stimulated by CT, mostly in pN- (Fig. 3G).

L -methionine (increased in stool gene patterns within patients >T1) tended to increase in the plasma of stage II-III patients post-CT (Supplementary Fig. S5B, C, right and left panel respectively). Krebs cycle metabolites were higher in stools of pN+ versus pN- and increased in plasma post- CT in pN+ (Fig. S5B). The iNOS regulator dimethylarginine was increased in the plasma post CT at later stages of BC, while L-arginine biosynthesis was found in stools of good prognosis BC (Supplementary Fig. S5D).

Altogether, significant changes occurred in the stool composition after 8 cycles of adjuvant anthracyclines and taxanes, expanding commensals associated with more favorable prognosis, the metabolic pathway of polyamines (L-Arg, L ornithine) and glutamine synthesis.

### Gut microbiota and chemotherapy-related side effects in CANTO

**Fig. 4:**
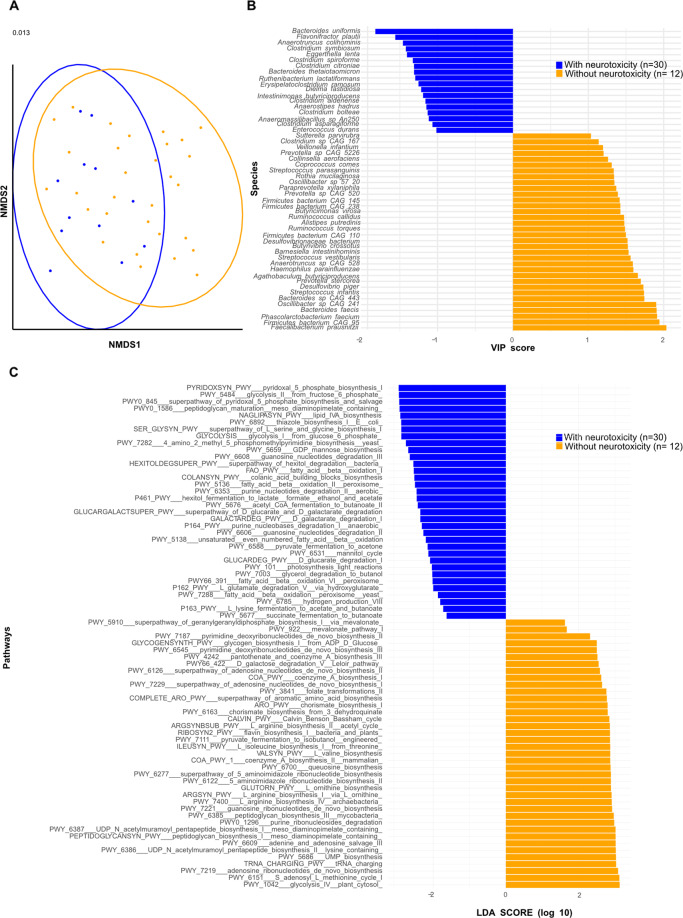
Metagenomics-based stool composition and gene related functional pathways are associated with neurological symptoms diagnosed at 12 months post-chemotherapy (CT). **A**. Beta-diversity ordination plot based on principal coordinate analysis of normalized and standardized data of post-CT fecal microbiota composition in patients with (yes, blue) and without (no, orange) long-term neurological toxicities evaluated 12 months after CT (*n* = 42). The *p* value is indicated at the top of the *Y* axis. **B** VIP scores showing the most discriminant species (only bacteria with prevalence >2.5% were considered) were obtained within partial least square discriminant analysis by differentiating between presence (yes, blue) and absence (no, orange) of neurotoxicity evaluated 12 months after CT (*n* = 42) in post-CT stools. **C** LEfSe method detected differential abundant metabolic pathways when discriminating between presence (yes, blue bars) vs absence (no, orange bars) of long-term neurotoxicity in 12 months post-CT samples.

The LEfSe method was used to compare the abundance of all bacterial clades pre- and post-CT according to the occurrence of toxicity at 12 months, considering neurological, gastrointestinal, rheumatological, or metabolic (BMI) disorders (Supplementary Table S3). The α-diversity of the gut microflora was not significantly different between patients with or without side effects pre-CT, but varied post-CT according to BMI, diarrhea and constipation (Supplementary Figs. S6A, C and  S7B, D). In univariate analysis, β-diversity post-CT significantly predicted neurological side effects (comprising paresthesia, peripheral sensory neuropathy, memory disorders, concentration defects) (Fig. 4A, *p* = 0.013, Supplementary Table S3), as well as overt weight gain (Supplementary Fig. S6), constipation, diarrhea or hot flashes, while rheumatological side effects were not significantly associated with the gut composition (Supplementary Fig. S7). 51 patients had neurological side effects at month 12 while 16 did not. Bacterial taxa with differential abundance between study groups complaining or not from neurological side effects were used as input for the LDA. The bacteria that were associated with neurological symptoms belonged to the Clostridiaceae family (i.e *C. symbosium, C. bolteae, C. spiriforme, C. aldenense*, *C. citroniae, C. asparagiforme* and *E. ramosum*) (Fig. 4B). They were also retained in the LEfSe model in pre-chemotherapy samples, as an internal validation (not shown). Most of these taxonomic species that were associated with neurological symptoms post-CT were clinically relevant for the dismal prognosis (as outlined Fig. 1 and Fig. 2A–D and Supplementary Figs. S2, S3, i.e, *C. bolteae, C. spiroforme, E. ramosum, E.coli, B. uniformis, B. thetaiotaomicron, Blautia wexlerae, Eggerthella lenta, Ruthenibacterium lactatiformans*).

Conversely, some of the bacteria underrepresented in patients who were less susceptible to develop neurological side effects also featured in the list of commensals associated with good prognosis and/or healthy status (*C.aerofaciens, C.comes, D.piger, R. torques, B. crossotus*). However, it is noteworthy that most of the bacteria associated with dismal prognosis that were dominant pre-CT (and disadvantaged by CT) were especially abundant in the stools of patients who did not suffer from weight gain (*C. bolteae, C. clostridioforme, C. aldenense, C. hathewayi, E. ramosum, E. coli, B. uniformis, Eisenbergiella tayi*, spp from the *Veillonella* genus and *Streptococci spp*.) (Supplementary Fig. S6, Supplementary Table S4).

The microbiome functional potential was analyzed at the genetic level in the MG data base of our CANTO cohort using the HUMAnN3 database [26]. Neurological side effects were associated with intestinal functional pathways involving mainly energy production with an enrichment in the glycolysis pathways, L-histidine degradation, fatty acid biosynthesis and beta-oxidation, as described for stools from patients with dismal prognosis. In contrast, microbial genes coding for enzymes involved in ribonucleotide de novo biosynthesis, polyamine biosynthesis and the GABA shunt were linked to neuroprotection (Fig. 4C).

### Relevance of the findings in gut humanized avatar mouse models of BC

**Fig. 5:**
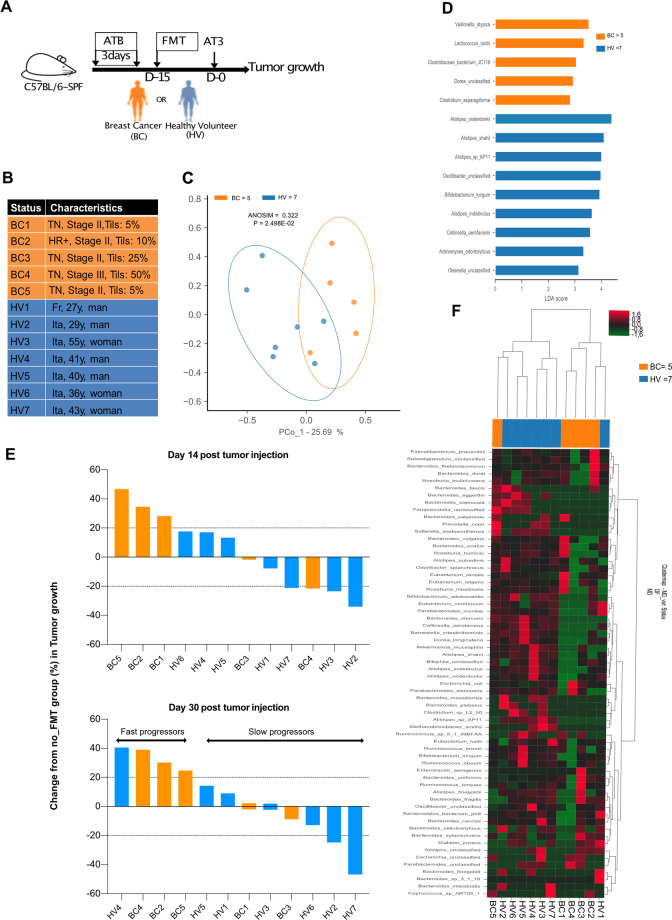
Gut humanized avatar AT3 tumor bearing mice mirrored patient prognosis. **A** Experimental setting of avatar mice. Fecal microbial transplantation (FMT) of feces from Healthy Volunteers (HV) or early breast cancer patients (BC) into AT3 tumor bearing C57BL/6 mice. Arrows details treatments. Each experiment contained 6 mice/groups for each FMT and each donor et was performed at least two times. **B** Details on clinical and pathological characteristics of human donors for FMT (five early BC and seven HV). **C** Beta-diversity ordination plot based on principal coordinate analysis of normalized and standardized data of donors’ fecal microbiota composition in BC (orange) and HV (blue) to analyze the most discriminant species in between the two donor groups. **D** LEfSe (Linear discriminant analysis of effect size) differentiating BC and HV, describing the 14 most discriminant species in descending order of importance in 5 early BC pre-CT versus 7 HV stools used for FMT in AT3 tumor bearing mice. **E** AT3 tumor size in each group of FMT at 2 time points, 14 days (Top panel) and 30 days (low panel) after tumor inoculation. Means are depicted for 5–6 mice/group. The tumor size changes (%) were compared with special pathogen free conditions (No FMT) at the same timepoint. A deviation of +20% compared with special pathogen free conditions (No FMT) was defined as “fast versus slow” progressors. Each experiment has been performed once for each donor with 12 FMT performed in a blinded manner. **F** Heatmap of the non-supervised hierarchical clustering of microbiota composition of stool samples across all 12 donors of FMT. Colors (from green to dark red) shows the row scaled relative abundance of each taxon across all samples.

In order to show a cause effect relationship between the gut composition and tumor aggressiveness, we took advantage of avatar mouse models, which are first conditioned by broad-spectrum antibiotics to eradicate the endogenous mouse microbiota and then “humanized” by fecal microbial transplantation (FMT). Such “humanized” immunocompetent C57BL/6 mice were inoculated with syngeneic transplantable AT3 BC cells as previously described [27] to investigate tumor development in the absence or presence of chemotherapy (Fig. 5A). We performed oral gavage with FMT using stools from seven independent HV and five BC patients at diagnosis (Fig. 5B) for whom the MG analyses were available (Fig. 5C, D). Fifteen days later, we challenged recipient avatar mice with a lethal dose of AT3 to follow BC growth kinetics in comparison with non FMT “eubiotic” mice reared in specific pathogen-free conditions. The LEfSe method was used to compare the abundance of all bacterial clades according to the clinical status (HV versus BC). Bacterial taxa with differential abundance between the two study groups were used as input for the linear discriminant analysis (LDA) to calculate an effect size (LEfSe analysis) that revealed a relative dominance of *Alistipes spp. (A. shahii* being associated with stage I BC (Supplementary Fig. S3) and *B. longum* and *C. aerofaciens* in PCoA, in HV compared with BC (Fig. 5C, D), in accordance with the contrast observed between HV and BC described in Fig. 2 and S3. When we monitored AT3 tumor progression, we observed two groups of stools that mediated opposite effects, one group that tended to mitigate natural tumor progression, called “slow progressors” (dominated by HV2, HV3, HV7) while the other group called “fast progressors” that tended to accelerate the natural kinetics (encompassing BC2, BC4, BC5, HV4-5) (Fig. 5E). The non-supervised hierarchical clustering of fecal MGS of the FMT donors could also segregate slow from fast progressing tumor bearers (Fig. 5F). A closer examination of the taxonomic species overrepresented in slow (versus fast) progressors revealed several members of the *Eubacterium* genus (*E. rectale, E. eligens), A. muciniphila*, Actinobacteria class (*B. longum, C. aerofaciens*) and *Alispites shahii*, which were already identified in patient stools from patient with stage I or N- BC pre-or post-CT. In contrast, fast progressors received fecal material containing MG species associated with dismal prognosis (such as *B. uniformis, B. xylanivolvens, B. intestinalis*, Fig. 5E).

**Fig. 6:**
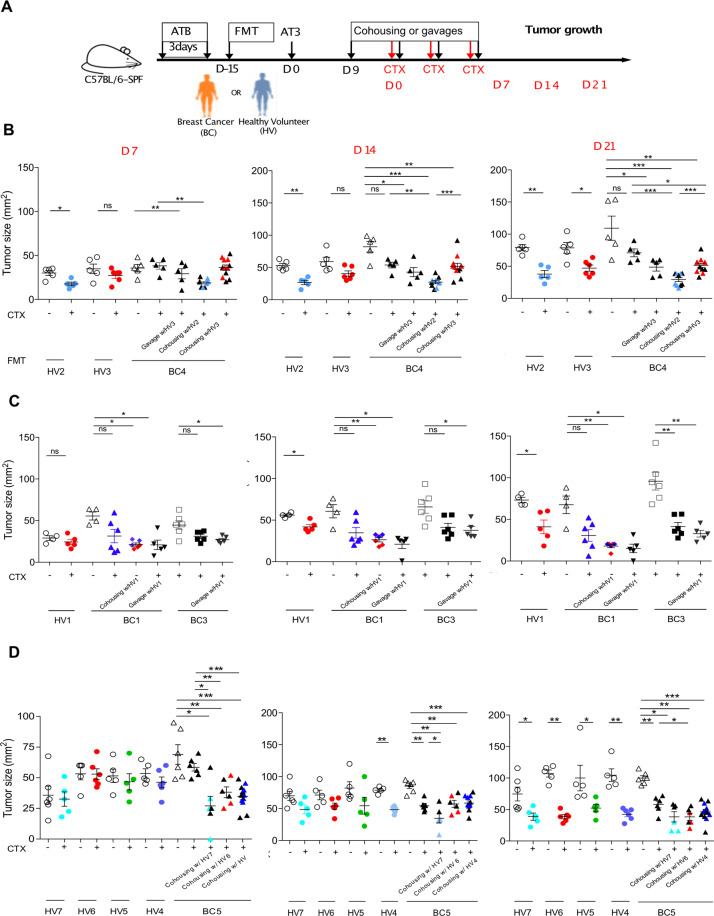
CTX-mediated tumoricidal activity is affected by the gut microbiota composition in avatar AT3 bearers. **A**. Experimental Setting. As in Fig. 5, FMT using stools from HV or early BC was performed following 3 days of ATB in specific pathogen-free (SPF) C57BL/6 mice. Two weeks later, AT3 breast tumors were subcutaneously inoculated. 9 days after, mice were randomly assigned to cohousing or FMT with different donors (HV or BC) and concomitantly treated with 4 intra peritoneal (i.p.) injections of cyclophosphamide (CTX). Tumor size was recorded for each mouse on days D7, D14, D21 after CTX starting. 5–6 mice/groups for each FMT and each donor. **B**–**D**. Means AT3 tumor sizes ± SEM at different timepoits (D7, D14, and D21 after CTX starting) showing the BC induced dysbiosis and compensation with FMT or cohousing with different HV (described in Fig. 5) resulting in rescued response to CTX (each color representing one donor, details on donors charactheristics are showed on Fig. 5B). Each experiment included 5–6 mice per group. ANOVA statistical analyses of means and SEM: *p < 0.05, **p < 0.01, ***p < 0.001, ns: non-significant.

Next, driven by previous reports showing that ATB negatively affect the efficacy of CT based on oxaliplatin [28] or cyclophosphamide (CTX) [7, 8], we addressed the question as to whether the microbiota composition could modulate the efficacy of CTX-based therapy in AT3 BC bearing “humanized” mice (Fig. 6A). “Humanization” by FMT from four BC patients and five HV (listed in Fig. 5B), demonstrated that CTX-mediated anticancer effects were affected by the intestinal microbiota. In the vast majority of mice bearing a human BC-associated gut microflora, the tumoricidal activity of CTX was barely significant, contrasting with mice that obtained an FMT from HV (Fig. 6B–D). Taking advantage of the coprophagic behavior of rodent, we treated mice “humanized” with BC feces and that were doomed to fail CTX-based chemotherapy by co-housing with “humanized mice” benefiting from a HV-derived microbiota or, alternatively, by oral gavage with HV-derived feces. In 3 independent experiments, HV-derived microbiota allowed to restore the CTX-mediated anticancer effects (Fig. 6B–D, Fig. 5B).

Altogether, these experiments demonstrate that the composition of the fecal microbiota influences the natural growth of BC (without therapy), as well as the response of BC to CTX-based CT. Thus, variations in intestinal microflora is not a mere correlate of BC progression but rather influence the course of the disease in a causative fashion.

## Discussion

To our knowledge, this study offers the first description of the gut microbiota repertoire defined by shotgun MG sequencing of fecal samples in early BC at diagnosis and its dynamics after adjuvant chemotherapy. The data presented herein suggest that (i) distinct commensals from the gut microbiome impact BC prognosis in patients and BC tumor aggressiveness in mice, (ii) chemotherapy can tilt the balance between favorable or unfavorable species, and iii) selected commensals may affect the probability of developing neurological side effects.

In contrast to previous reports focusing in metastatic melanoma or advanced NSCLC receiving PD-1-targeting immune checkpoint blockade (ICB), α diversity was not predictive of favorable BC prognosis, nor of side effects [12, 14, 29]. In contrast, β diversity of the gut microbiota was associated with tumor grading (but not breast cancer subtype), LN involvement and staging, as well as with neurological side effects at 12 months. At diagnosis (and regardless of chemotherapy), fecal composition containing a relative dominance of the trio of *C. bolteae, C. asparagiforme*, and *B. uniformis* was associated with axillary lymph node invasion, as previously described for resistance to ICBs in advanced NSCLC and kidney cancers [19, 27]. In contrast, *E. rectale, M. smithii, C. comes or C. catus*., and *Actinobacteria (C. aerofaciens)* that were associated with the HV status and dominant in feces of N- and/or stage I BC patients also correlated with favorable prognosis of patients with kidney cancer or melanoma treated with ICBs [12, 19]. Of note, *C. aerofaciens* was also retrieved by LEfSe analysis of the “humanized” gut from AT3 bearing mice exhibiting slower tumor progression (Fig. 5). Our data are in line with a pioneering shot gun MG analysis performed on stools from 18 premenopausal and 44 post-menopausal women diagnosed with BC [30]. This latter study indicated significant differences between BC and healthy controls post-menopause, with increased α diversity and pro-inflammatory *Enterobacteriaceae* members (*E. coli, Klebsiella* spp), and a concomitant decrease in *Eubacterium eligens* in BC patients (correlating with decreased tumor-infiltrating lymphocytes, TILs) compared with HVs.

Moreover, HR + BC, which constitute the largest group in our study, are not expected to be under strong T cell-based immunosurveillance. HR + BC are poorly infiltrated with TILs. Of note, our work did not reveal any significant correlation between the gut microbiota and the density of TILs (not shown). One might speculate that the intestinal ecosystem of women doomed to develop this BC subtype might be relatively poor in commensals associated with a strong immune tonus, such as *A. muciniphila, B. fragilis, E. hirae, Bifidobacteria spp. Eubacteriaceae family members*. Supporting this notion*, A. muciniphila* was found associated with small tumor size (pT1) BC in our study. More than 55% women with BC lacked detectable *A. muciniphila*, in line with the association of BC with type 2 diabetes and high BMI, two conditions that are also associated with the absence of *A. muciniphila* [31].

Importantly, we found that chemotherapy drastically shifted the microbiome composition, tilting the balance towards favorable commensals in a subset of patients. Hence, in two BC subcohorts, we observed that several bacterial spp. associated with ATB uptake or resistance to ICB (i.e Clostridia such as *C. hathewayi, C. clostridioforme, C. symbosium, C. aldenense, C. citroniae, E. ramosum*, as well as *Veillonella* spp, and *Eisenbergiella massiliensis* or *E. tayi*) which were associated with N + status at diagnosis and neurological side effects, became less abundant after adjuvant or neoadjuvant treatment with anthracylines and taxanes. In contrast, CT favored the colonization or residence of favorable commensals such as *Methanobrevibacter smithii, D. formicigenerans, Blautia obeum,* or *R. torques*. Importantly, the gut humanized avatar AT3 bearers that were subjected to FMT containing species from the *Eubacterium* genus (*E. rectale, E. eligens, E. ventriosum)* or *C. aerofaciens* exhibited a reduced tumor growth rate compared with eubiotic control mice. A pioneering study [32] focusing on the possible involvement of microbiota changes in the beneficial effects of metronomic (versus standard dosing of) capecitabine in 15 metastatic HER2 negative BC described differences in commensalism between the two CT regimens and the association of *Slackia* and *Blautia obeum* with poor and favorable clinical outcome, respectively. Therefore, it will be important to validate the capacity of specific chemotherapeutic agents to eliminate unfavorable bacteria or to expand favorable commensals in future clinical studies.

Very few reports previously evocated a potential impact of the gut microbiome on BC risk or prognosis [33]. One study investigated stools from 31 early BC patients by real-time qPCR of genes encoding for distinct bacterial families or species-related 16S rRNA [34]. The authors of this study concluded that *Clostridium leptum* and *C. coccoides* clusters, which express β-glucuronidases favoring the reabsorption of free estrogen, were enriched in patients with stage II-III (as opposed to stage I) BC [34]. Our study supports the previously described deleterious role of *Clostridiacae* family members (*C. citroniae, C. bolteae, C. clostridioforme, C. symbosium, C. aldenese, C. hathewayi, C.asparagiforme*) across various malignancies (kidney, lung, and breast cancers) and therapies (ICBs, anthracyclines, taxanes, CTX).

Mechanistically, an earlier report pioneered the idea that microbiota can dictate the prognosis of extraintestinal ovarian and breast cancers via an effect on Toll-like receptor 5 (TLR5) [6]. TLR5 recognition of flagellin-expressing commensals may favor systemic tumor -promoting inflammation and malignant progression of BC. Thus, TLR5 signaling drives recirculation of IL-6, mobilizes of myeloid suppressor cells and elicits immunosuppressive galectin-1-producing γδT cells [6].

More importantly, overt commensal dysbiosis might cause systemic inflammation that induced collagen deposition and accelerated fibrosis of the normal and tumor mammary gland through infiltration of myeloid cells [35]. Besides promoting systemic inflammation, gut dysbiosis may alter whole body metabolism. Zhu et al. explored functional features of the gut microbiota in pre- and post-menopausal control in BC females, and reported that amino acid transport and nucleotide synthesis dominated the KEGG modules in pre-menopause, while LPS and beta-oxidation were mostly found post-menopause [30]. Of note, our study pointed to the overrepresentation of lipid beta-oxidation modules in BC patients prone to develop neurological side effects. Conversely, our study suggested an association between protection from taxane-induced neurological side effects and the overrepresentation of purinergic pathways in the gut microbiota. These latter finding might be interpreted to suggest that increased production of the neuroprotective purine adenosine (which reportedly favors neurotransmission [36], affects neuro-immunity [37], maintains the blood-brain barrier [38] and avoids neurodegeneration [39–41] through effects on A1, A2A, A2B, and A3 adenosine receptors [42]) might prevent the taxane-induced neuropathy by direct effects on neural circuitries. Moreover, the metabolic pathway of polyamines is associated with better prognosis and neuroprotection in our cohort. Polyamines constitute major products of the colonic microbiota, as shown after oral administration of the immunogenic commensal *A. muciniphila* [43]. Polyamines can improve the intestinal barrier function [44] and cancer immune-surveillance [45].

Finally, CT stimulate intestinal ketogenesis. Since ketone bodies favor immunosurveillance [46], this effect may contribute to the anticancer effects of CT.

Altogether, the findings reported in this study may have important consequences for the future clinical management of early BC patients. *First*, monitoring the presence and abundance of favorable *versus* unfavorable commensals may be important to predict the efficacy of adjuvant or neoadjuvant chemotherapy. *Second*, compensatory therapies aimed at providing immunogenic commensals may turn cold HR^+^ BC into TIL-enriched tumor microenvironment amenable to ICBs and/or more sensitive to chemotherapies. Such an effect has been achieved with live biotherapeutics in preclinical models [9, 27, 47]. Supporting the notion that metabolism can be kept in check by commensals, a preparation of oral pre-and probiotics (containing *E. halii, A. muciniphila,* and inulin) reduced postprandial glucose levels in type 2 diabetes patients in a randomized double blind placebo controlled trial [48], confirming earlier trials [49]. Thus, it is possible to treat human disease by intervening on the gut microbiota. *Third*, diet interventions may represent yet another way to influence the gut microbiota. While promising approaches such as fasting or ketogenic [46] diets have direct cell autonomous or indirect immune effects on cancer, it remains to be determined whether fasting-induced microbial shifts may participate in the immune control of HR^+^ BC [24]. Thus, future studies must determine the best strategy to manipulate the intestinal microbiota to improve the clinical prognosis and quality of life of BC patients.

## Materials and methods

### Clinical study

We enrolled patients from CANTO trial (for CANcer TOxicities NCT01993498), a prospective clinical cohort including non-metastatic breast cancer (BC). The CANTO study aims at performing a long-term follow-up of 13 250 women treated for BC over a period of ten years in order to quantify and prevent chronic toxicities related to treatment (surgery, radiation therapy, chemo-hormono-therapy). The present analysis focuses on a first set of 9595 pts enrolled from 2012 to 2017 with sufficient follow-up and mature data in 2019, of which 76 were included in the biological microbiota sub-study (refer to Consort diagram, Supplementary Fig. S1). Patients stools were collected before and after chemotherapy (adjuvant or neoadjuvant) at home, in kits including an anaerobic generator (Biomerieux). Samples were frozen 4–24 h later at −80 °C at Gustave Roussy Cancer Campus in plastic tubes (Plastic vessel by 1000-Sarstedt) with or without RNA later. Plasma samples were collected for 29 BC females: 26 samples before chemotherapy and 29 after chemotherapy. In parallel, clinical data were collected prospectively at three time points: at diagnosis (baseline); 3–6 months after the end of primary treatment (i.e., primary surgery, chemotherapy, or radiotherapy, whichever came last), and then 1 year post primary treatment. Antihuman epidermal growth factor receptor (HER2) therapy and hormonal therapy could be ongoing, if indicated. Toxicity data were collected at 3–6 months and 1 year after the end of primary treatment. Clinical characteristics and toxicities are described in supplemental tables (Supplementary Tables 1, 2, and 3). CANTO is coordinated by UNICANCER, the National Cooperative Group of French Cancer Centers. The study was approved by the national regulatory authorities and ethics committee (ID-RCB: 2011-A01095-36, 11-039). All patients enrolled in the study provided written informed consent, including consent for the biological data collection.

Furthermore, we used the feces of 54 Italian HV from Instituto Nazionale dei Tumori center, whose feces were harvested according to the same procedures and analyzed together with 282 HV-derived samples selected from referenced public metagenomes.

### Metagenomics analysis

Total fecal DNA was extracted as described [50, 51] and sequenced using ion-proton technology (ThermoFisher) resulting in 22.7 ± 0.9 million (mean ± SD) single-end short reads of 150-base-long single-end reads as a mean. Reads were cleaned using [52]. Alien Trimmer in order (i) to remove resilient sequencing adapters and (ii) to trim low quality nucleotides at the 3′ side using a quality and length cut-off of 20 and 45 bp, respectively. Cleaned reads were subsequently filtered from human and other possible food contaminant DNA (using Human genome RCh37-p10, Bos Taurus and Arabidopsis thaliana and an identity score threshold of 97%). MetaPhlAn3 pipelines were used to profile fastq files deriving from shotgun sequencing in order to describe the bacterial species present in each sample [53]. These pipelines are based on a gene-reference database, thus protein-coding gene markers deriving from the collection of the +100k bacterial genomes (which we especially focused on) deposited in NCBI or ENA archives. With few gene markers available for each bacterial species (ranging from 50 to 400) it was thus possible to ascertain their presence and relative abundance (expressed in the interval 0–100%) within assembled shotgun samples. For the MetaOMineR analyses the gene abundance profiling was performed using the 9.9 million gene integrated reference catalog of the human microbiome [54]. Filtered high-quality reads were mapped with an identity threshold of 95% to the 9.9 million-gene catalog using [55]. Bowtie 2 included in METEOR software [56]. The gene abundance profiling table was generated by means of a two-step procedure using METEOR. The gene abundance table was processed for rarefaction and normalization and further analysis using the MetaOMineR package [57]. The gene abundance table was rarefied to 13 million reads per sample (a threshold chosen to include all samples, but 1 with 12.5 million reads) by random sampling of 13 million mapped reads without replacement. The resulting rarefied gene abundance table was normalized according to the FPKM strategy (normalization by the gene size and the number of total mapped reads reported in frequency) to give the gene abundance profile table. Metagenomic species (MGS) are co-abundant gene groups with more than 500 genes corresponding to microbial species. 1436 MGS were clustered from 1267 human gut microbiome samples used to construct the 9.9 million-gene catalog [54], as described [58]. Differentially abundant MGS between different patients’ groups were selected using the Wilcoxon test (*p* < 0.05). Microbial gene richness (gene count) was calculated by counting the number of genes that were detected at least once in a given sample, using the average number of genes counted in ten independent rarefaction experiments. MGS richness (MGS count) was calculated directly from the MGS abundance matrix. For the MetaPhlAn3 analyses fastq files were cleaned/filtered as described above and underwent an additional filtering for possible human contaminants (reference database GRCh37/hg19) and contextual quality control using KneadData. This wrapper entangles Bowtie2 (“-very-sensitive” and “-dovetail” settings) to rule out contaminant sequences and Trimmomatic (sliding window 20, min-length 50) to exclude low-quality reads. Filtered reads underwent MetaPhlAn3 pipeline (default settings) for unambiguous taxonomic classification and to generate a table of relative abundances for bacterial, archael, eukaryotic species. Only taxa that were present in at least 5% of all samples were kept for downstream analysis. Measurements of α diversity (within sample diversity) in terms of Shannon index, Simpson index, and richness were calculated at species level using the diversity function in the R environment. Exploratory analysis of β-diversity (between sample diversity) was calculated using the Bray-Curtis measure of dissimilarity and represented in PCoA.

Supervised PLS-DA and the subsequent VIP were used to find out the most discriminant bacterial species among the cohorts, having a prevalence cutoff >=5%. Two-tailed Mann–Whitney *U* and Kruskall Wallis tests were employed using the function in the R environment to assess significance for pairwise or multiple comparisons, respectively, considering a *P* value ≤ 0.05. Longitudinal analysis differences were assessed by Mann–Whitney *U* test. All *P* values underwent a Benjamini-Hochberg two-stages false detection rate at 10%. Gene-family and pathway-relative abundances were generated by HUMAnN3 [53]. Univariate analyses were performed using LEfSe [59] to estimate the effect size of features that were statistically significant among groups. The biomarkers with the highest effect sizes were reported and discussed. Cross-correlation Pearson matrices for network analysis (metric = Bray–Curtis, method = complete linkage) were generated and visualized with Gephi version 0.9.2.

### Metabolomics analysis

#### Plasma sample preparation

Plasma samples (50 μL) were mixed with 500 µL of the of ice-cold extraction mixture, allowing protein precipitation and metabolites extraction, then vortexed and centrifuged (10 min at 15000 *g*, 4 °C). Supernatants were collected, split in 4 fractions, and treated according to the protocols described in Viltard et al. [60]. Briefly, upper phase of supernatant was split in three parts: 150 µL were used for GC-MS experiment in injection vial, 40 µL were used for the SCFA (Short Chain Fatty Acids) HPLC-MS method, and 120 µL were used for others UHPLC-MS experimentations. The 4th fraction together with the sample pellet were re-extracted with an equal volume of 2% SSA (in meOH), vortexed and centrifuged (10 min at 15000 g, 4 °C) for polyamines detection.

#### Widely-targeted analysis of intracellular metabolites

*GC/MS*. GC-MS/MS method was performed on a 7890B gas chromatography (Agilent Technologies, Waldbronn, Germany) coupled to a triple quadrupole 7000C (Agilent Technologies, Waldbronn, Germany) equipped with a High sensitivity electronic impact source operating in positive mode [60].

#### Targeted analysis of bile acids

Targeted analysis was performed on a RRLC 1260 system (Agilent Technologies, Waldbronn, Germany) coupled to a QTRAP 6500+ (Sciex) equipped with an electrospray source operating in negative mode. Gas temperature was set to 450 °C, with ion source gas 1 and 2 set to 30 and 70, respectively [60].

#### Targeted analysis of polyamines

Targeted analysis was performed on a RRLC 1260 system (Agilent Technologies, Waldbronn, Germany) coupled to a Triple Quadrupole 6410 (Agilent Technologies) equipped with an electrospray source operating in positive mode. The gas temperature was set to 350 °C with a gas flow of 12 l/min. The capillary voltage was set to 3.5 kV [60].

#### Targeted analysis of short chain fatty acid

Targeted analysis was performed on a RRLC 1260 system (Agilent Technologies, Waldbronn, Germany) coupled to a Triple Quadrupole 6410 (Agilent Technologies) equipped with an electrospray source operating in negative mode. Gas temperature was set to 350 °C with a gas flow of 12 L/min. Capillary voltage was set to 4.0 kV [60].

#### Pseudo-targeted analysis of intracellular metabolites

The profiling experiment was performed with a Dionex Ultimate 3000 UHPLC system (Thermo Scientific) coupled to a Q-Exactive (Thermo Scientific) equipped with an electrospray source operating in both positive and negative mode and full scan mode from 100 to 1200 m/z. The Q-Exactive parameters were: sheath gas flow rate 55 a.u., auxiliary gas flow rate 15 a.u., spray voltage 3.3 kV, capillary temperature 300 °C, S-Lens RF level 55 V. The mass spectrometer was calibrated with sodium acetate solution dedicated to low mass calibration [60].

#### Statistical analysis

All targeted and pseudo-targeted treated data were merged and cleaned with a dedicated R (version 3.4) package (@Github/Kroemerlab/GRMeta). Calculations and statistical tests were performed using R v3.4. Wilcoxon–Mann–Whitney test was used to assess differences in concentration between two different groups. Data representation was performed with softwares R v3.6 and Rstudio v1.2.1335 using tidyverse, dplyr, ggplot2, ggpubr, complexheatmap, and corrplot packages.

### Experimental tumor model and treatments

- *Mice*: All animal experiments were carried out in compliance with French and European laws and regulations. The local institutional animal ethics board and French Ministère de la Recherche approved all mouse experiments (permission numbers: 2016-049-4646, 2018-078-17530). Experiments were performed in accordance with Government and institutional guidelines and regulations. Female C57BL/6 were purchased from Harlan (France). Mice were used between 7 and 12 weeks of age. All mouse experiments were performed at the animal facility in Gustave Roussy Cancer Campus where animals were housed in specific pathogen-free conditions.
- *Tumor models and CTX treatment*: C57BL/6-derived AT-3 mouse breast tumor cell line was processed as previously described [61]. To examine s.c. tumor growth, mice were inoculated subcutaneously on the right flank with 5 × 10^5^ of cells and tumor size monitored. Once tumors were established (20–40 mm^2^). Mice were injected weekly i.p with 100 mg/kg of cyclophosphamide (CTX, Endoxan, Baxter provided by Gustave Roussy) for three injections or equivalent volume of PBS. Mice were randomized based on their tumor sizes to homogenize the groups.
  Mice were excluded of the analysis if the reached the endpoint established by ethical committee such as tumor size or tumor necrosis.
- *Antibiotics:* Mice were treated with an antibiotic cocktail (ATB) containing ampicillin (1 mg/ml), streptomycin (5 mg/ml), and colistin (1 mg/ml) (Sigma-Aldrich), in the drinking water of mice. Antibiotic activity was confirmed by cultivating fecal pellets resuspended in BHI +15% glycerol at 0.1 g/ml on COS (Columbia Agar with 5% Sheep Blood) plates for 48 h at 37 °C in aerobic and anaerobic conditions. ATB were given during 3 days and FMT was performed the next day.
- *FMT*: FMT was performed by thawing fecal material. Two hundred µL of the suspension was then transferred by oral gavage into ATB pretreated recipient. In addition, another 100 µL was applied on the fur of each animal. Two weeks after FMT, tumor cells were injected subcutaneously. Mice were treated with CTX or PBS and randomly assigned to +/− oral gavage of fecal samples from HV into humanized mice with BC patients FMT. The experimenter was blinded for the healthy volunteer or breast cancer status.
- *Cohousing experiments*: Two weeks after FMT, tumor cells were injected subcutaneously. Mice were treated with CTX or PBS, then randomly assigned to +/− cohousing with humanized mice with HV or BC.

The experimenter was blinded to the healthy or breast cancer status of the patients. Each experiment included 5-6 mice per group. Data are representative of at least two independent experiments.

### Statistical analysis

Data analyses and representations were performed either with the statistical environment, Microsoft Excel or Prism 6 (GraphPad).

The Mann–Whitney *U* test was used to compare two independent groups, whereas nonparametric Kruskal–Wallis tests were implemented for multiple groups. For comparisons between patient groups, the Mann–Whitney *U* test *P* value was used.

All tests were two-sided and *P* values < 0.05 were considered to be statistically significant.

Data from in vivo experiments were expressed as the mean ± standard error of the mean (SEM). Data are representative of at least two independent experiments.

## Supplementary information

Supplementary Figure 1

Supplementary Figure 2

Supplementary Figure 3

Supplementary Figure 4

Supplementary Figure 5

Supplementary Figure 6

Supplementary Figure 7

Supplementary Table 1

Supplementary Table 2

Supplementary Table 3

Supplementary Figure and Table legends

## Data Availability

Raw data for the metagenomes newly sequenced in this study are available in NCBI-SRA under the BioProject PRJNA718520.
