# Supplementary figures and images for: Intestinal microbiota influences clinical outcome and side effects of early breast cancer treatment
Source: Cell Death Differ. 2021 May 7;28(9):2778–96. doi: 10.1038/s41418-021-00784-1 (PMC8408230; doi:10.1038/s41418-021-00784-1)

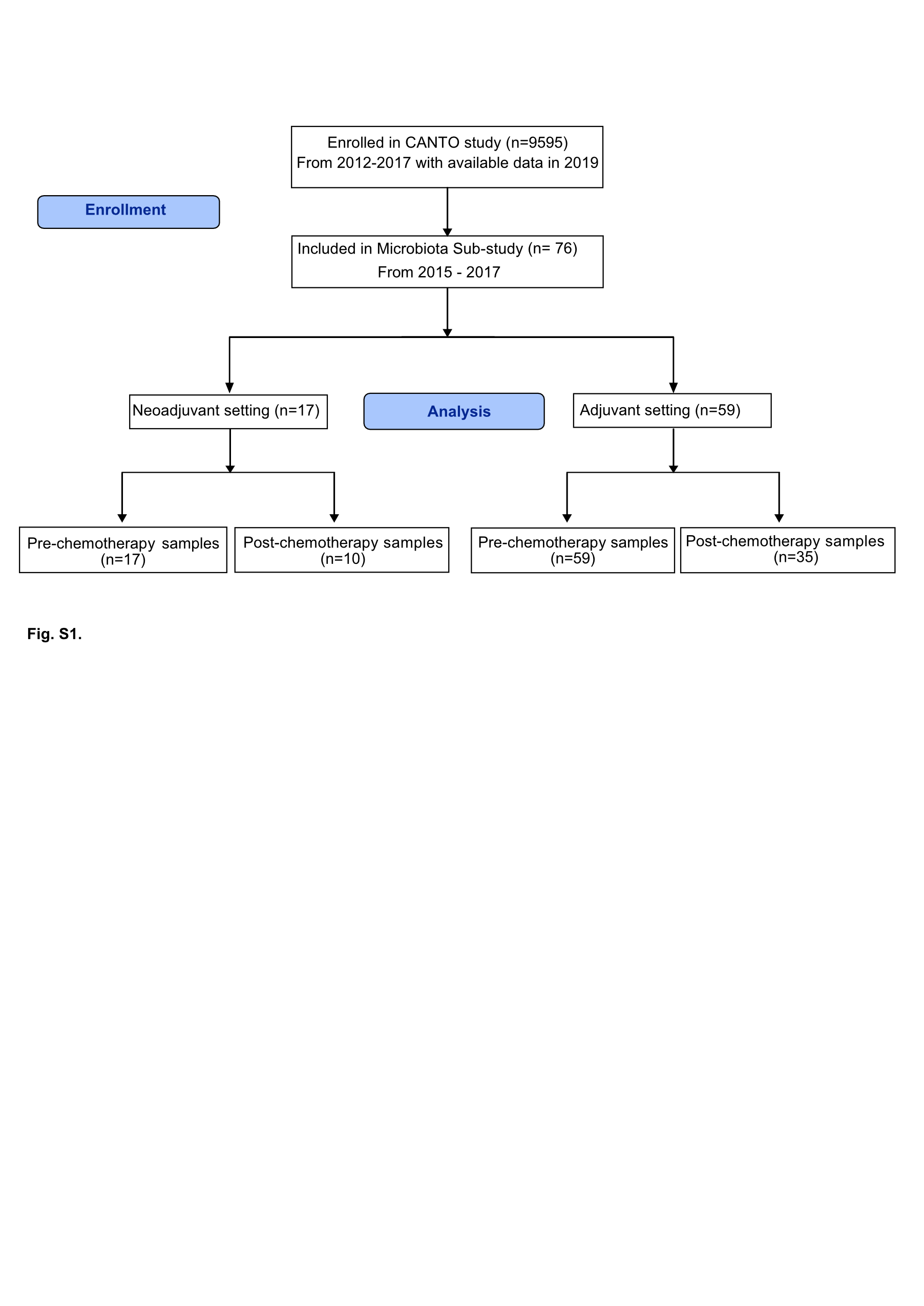

Supplement: Supplementary file 1 — Supplementary Figure 1 [file 41418_2021_784_MOESM1_ESM.png]

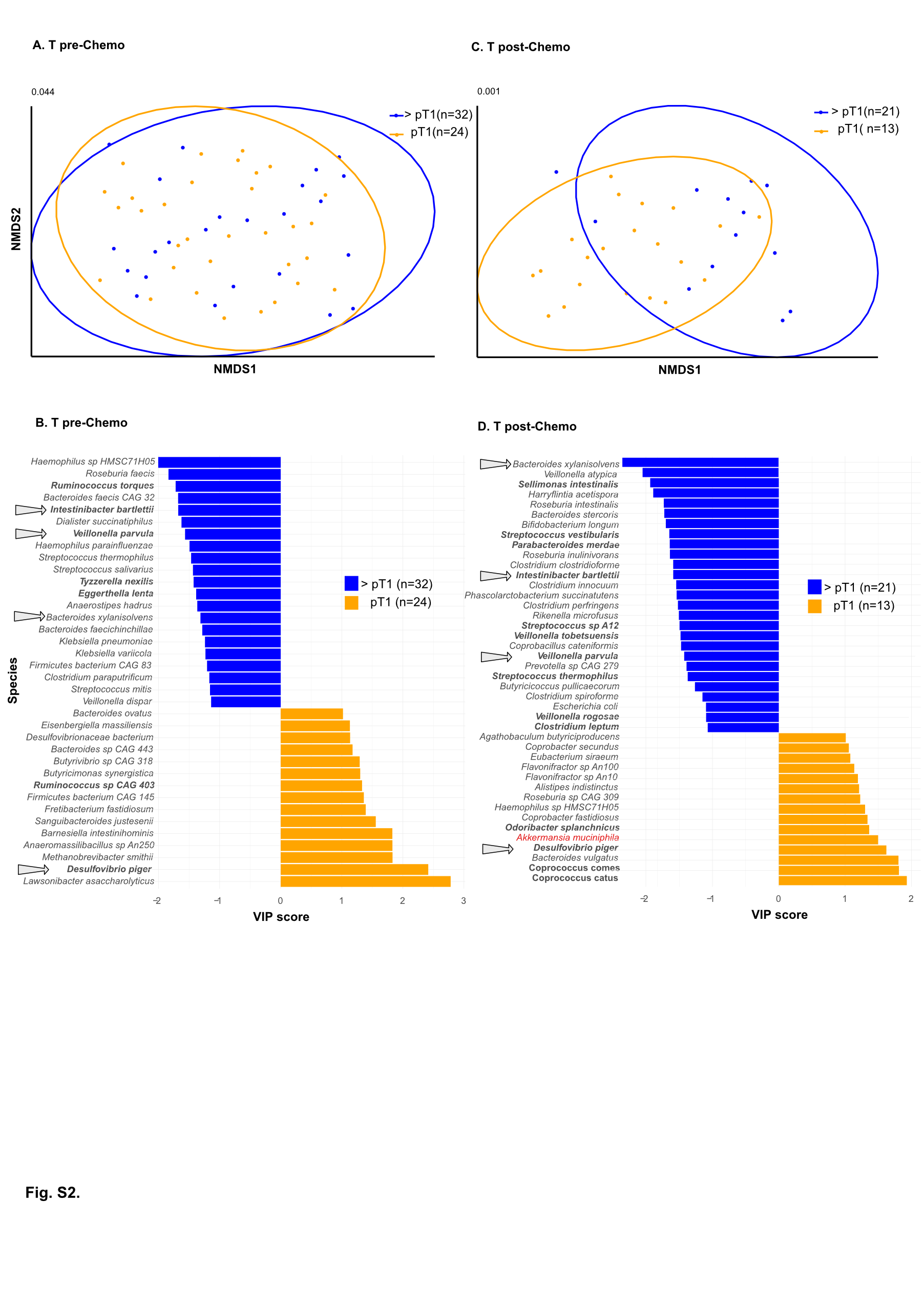

Supplement: Supplementary file 2 — Supplementary Figure 2 [file 41418_2021_784_MOESM2_ESM.png]

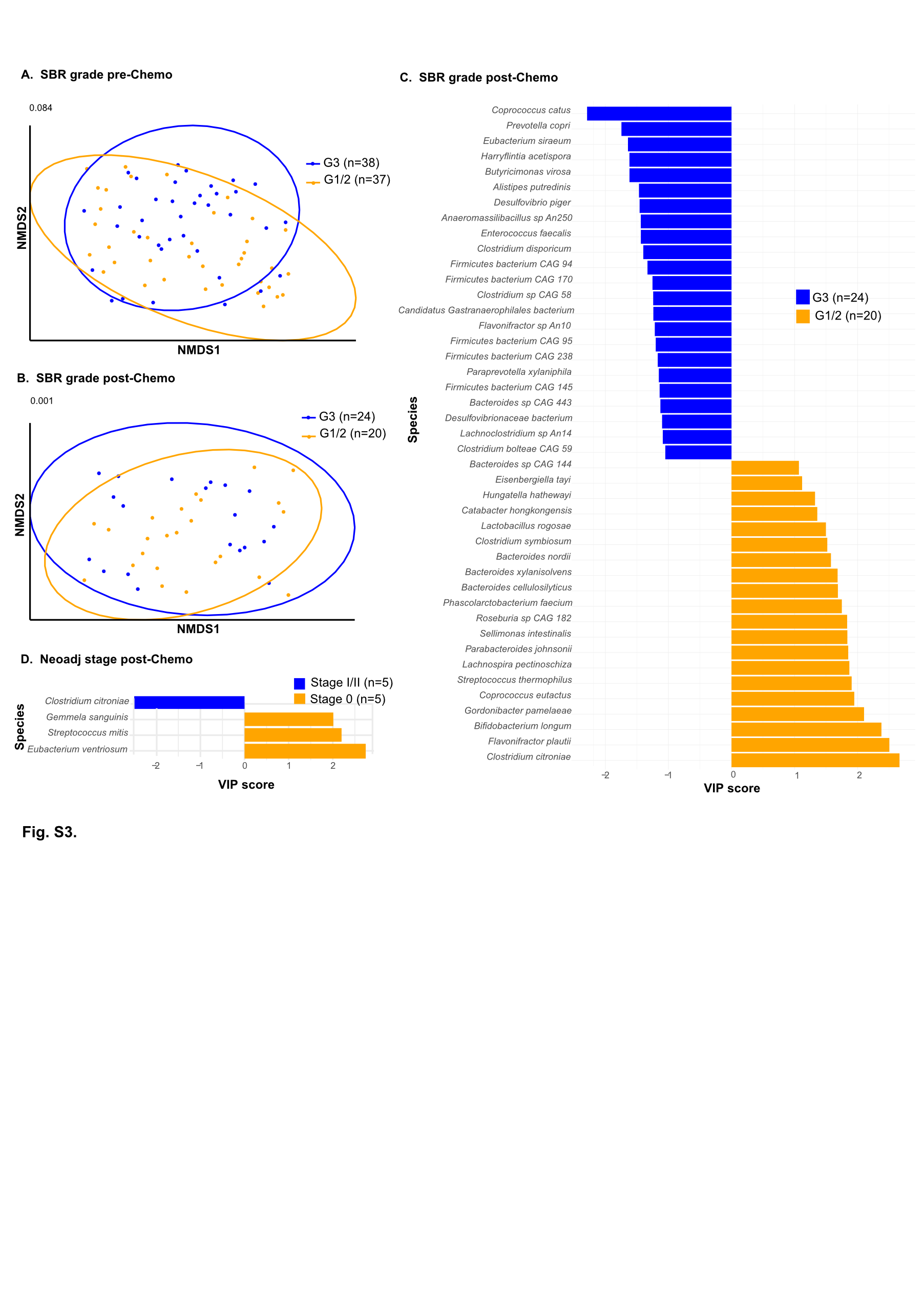

Supplement: Supplementary file 3 — Supplementary Figure 3 [file 41418_2021_784_MOESM3_ESM.png]

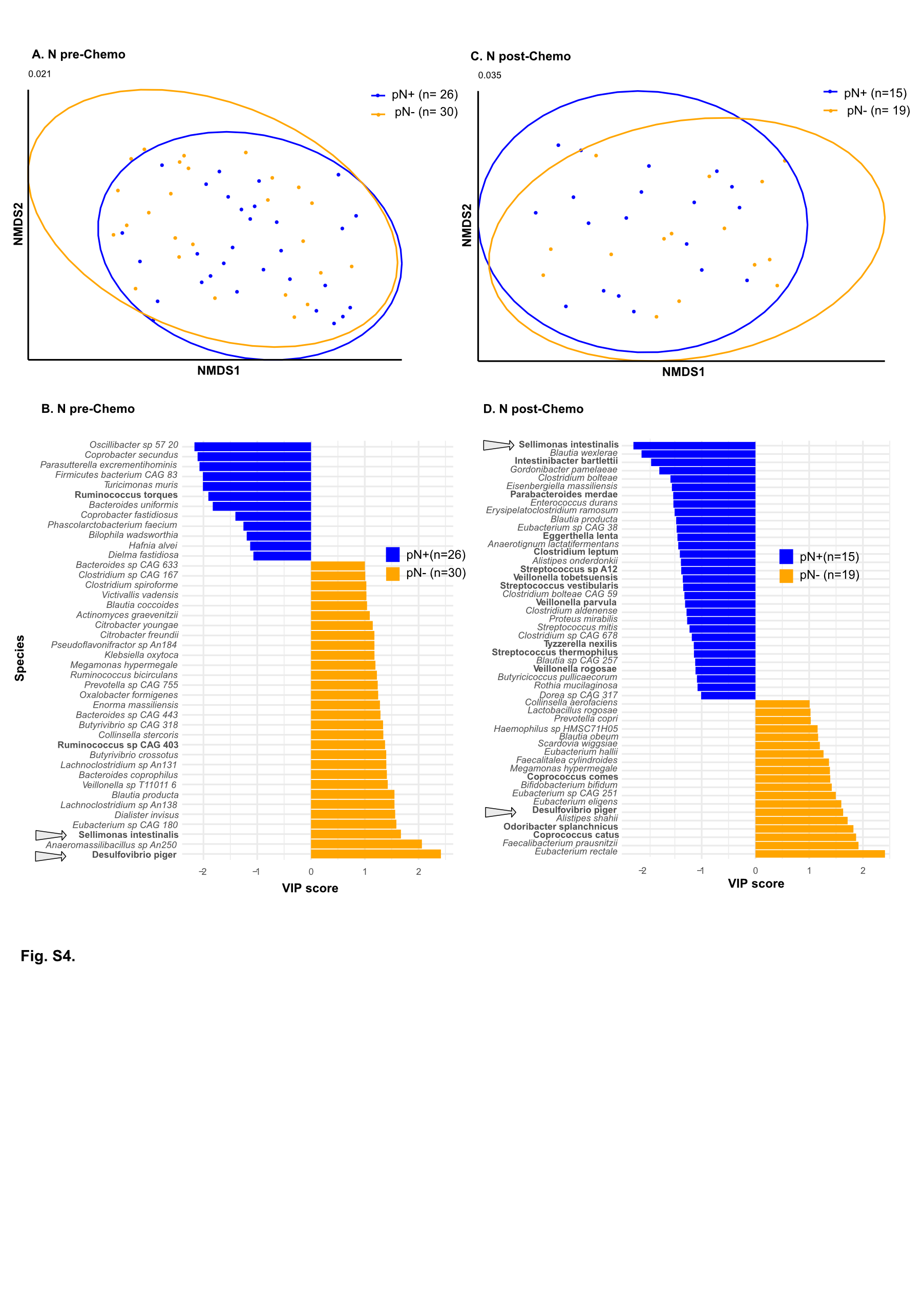

Supplement: Supplementary file 4 — Supplementary Figure 4 [file 41418_2021_784_MOESM4_ESM.png]

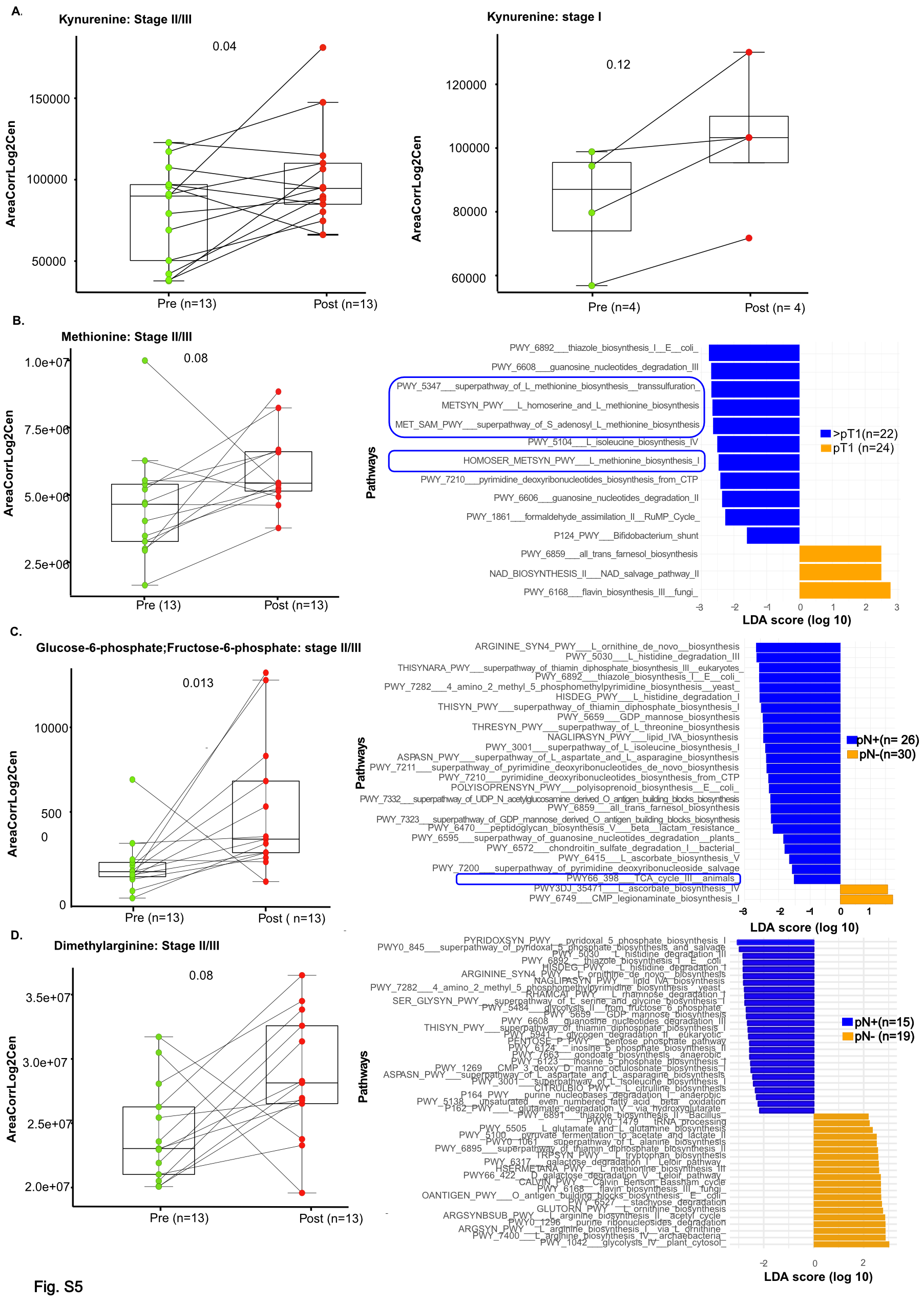

Supplement: Supplementary file 5 — Supplementary Figure 5 [file 41418_2021_784_MOESM5_ESM.png]

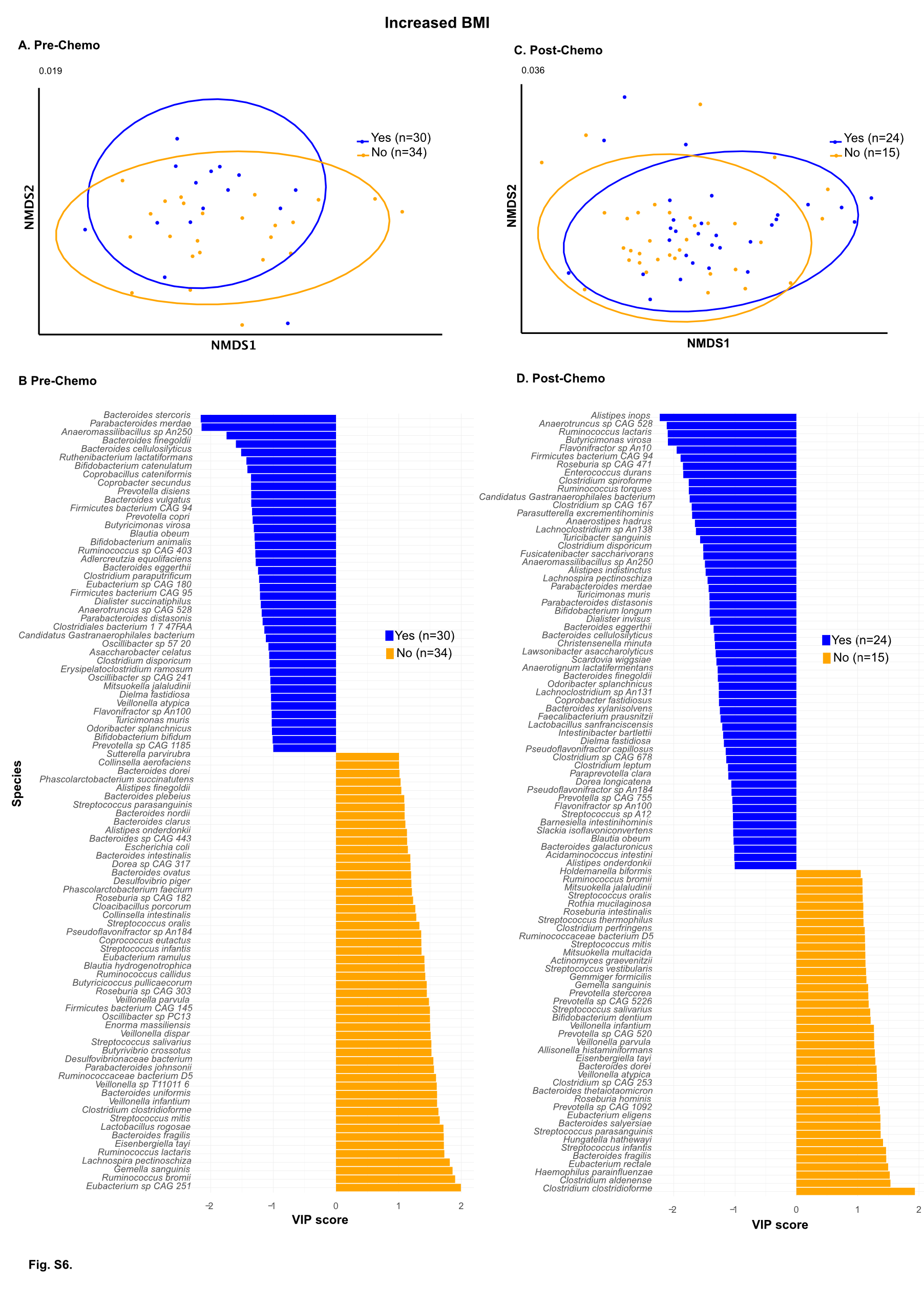

Supplement: Supplementary file 6 — Supplementary Figure 6 [file 41418_2021_784_MOESM6_ESM.png]

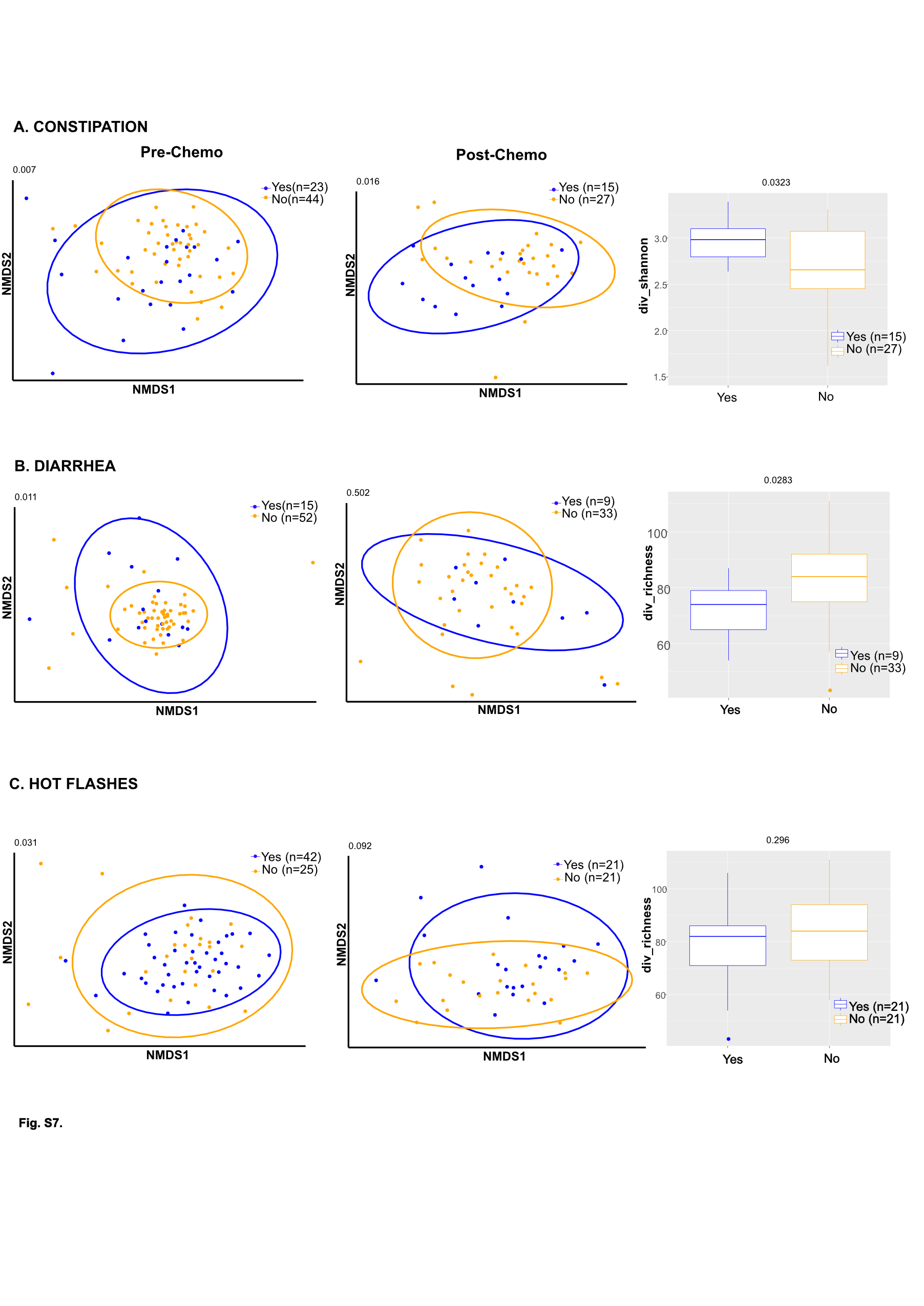

Supplement: Supplementary file 7 — Supplementary Figure 7 [file 41418_2021_784_MOESM7_ESM.png]
