# Supplementary material for: Intestinal microbiota influences clinical outcome and side effects of early breast cancer treatment
Source: Cell Death Differ. 2021 May 7;28(9):2778–96. doi: 10.1038/s41418-021-00784-1 (PMC8408230; doi:10.1038/s41418-021-00784-1)
Supplement: Supplementary file 8 — Supplementary Table 1 [file 41418_2021_784_MOESM8_ESM.docx]

| Table S1. Clinical characteristics. |  |  |
| --- | --- | --- |
|  | Pre-CT  n=76 | Post-CT  n=45 |
| **Age, years, median (range)** | 52.0 [46.0-62.0] | 52.0 [46.0-65.0] |
| **BMI Baseline, median (range)** | 26.9 [23.8-31.7] | 27.1 [22.7-30.2] |
| **BMI Baseline, no (%)** |  |  |
| <18.5 | 2 (2.63) | 1 (2.22) |
| [18.5;25[ | 24 (31.58) | 14 (31.11) |
| [25;30[ | 28 (36.84) | 17 (37.78) |
| >30 | 22 (28.95) | 13 (28.89) |
| **BMI at Month 12, median (range)** | 26.8 [23.2-32.3] | 26.5 [22.5-31.6] |
| **BMI at Month 12, no (%)** |  |  |
| <18.5 | 1 (1.56) | 0 |
| [18.5;25[ | 23 (35.94) | 15 (38.46) |
| [25;30[ | 18 (28.13) | 12 (30.77) |
| >30 | 22 (34.38) | 12 (30.77) |
| Missing | 12 | 6 |
| **Histologic grade (SBR), no (%)** |  |  |
| Grade 1 | 3(4.00) | 2(4.55) |
| Grade 2 | 34(45.33) | 18(40.91) |
| Grade 3 | 38(50.67) | 24(54.55) |
| Missing | 1 | 1 |
| **Tumor size (pT), no (%)** |  |  |
| *Adjuvant setting* |  |  |
| pT1 | 32(57.14) | 21(61.76) |
| >pT1 | 24(42.86) | 13(38.24) |
| Missing | 3 | 1 |
| *Neo-adjuvant setting* |  |  |
| pT0 | 9(52.94) | 5(50.00) |
| pT1 | 6(35.29) | 3(30.00) |
| >pT1 | 2(11.76) | 2(20.00) |
| **Pathological lymph node infiltration (pN), no (%)** |  |  |
| *Adjuvant setting* |  |  |
| pN+ | 26(46.43) | 15(44.12) |
| pN- | 30(53.57) | 19(55.88) |
| Missing | 3 | 1 |
| *Neo-adjuvant setting* |  |  |
| pN+ | 4(23.53) | 1(10.00) |
| pN- | 13(76.47) | 9(90.00) |
| **Pathological stage (in place of AJCC stage), no (%)** |  |  |
| *Adjuvant setting* |  |  |
| STAGE I | 21(37.50) | 14(41.18) |
| STAGE II | 27(48.21) | 14(41.18) |
| STAGE III | 8(14.29) | 6(17.65) |
| Missing | 3 | 1 |
| *Neo-adjuvant setting* |  |  |
| STAGE 0 | 8(47.06) | 5(50.00) |
| STAGE I | 4(23.53) | 3(30.00) |
| STAGE II | 5(29.41) | 2(20.00) |
| **Molecular subtypes, no (%)** |  |  |
| HR+/HER2- | 32(42.11) | 18(40.00) |
| HR±/HER2+ | 26(34.21) | 16(35.56) |
| HR-/HER2- | 18(23.68) | 11(24.44) |
| **(Neo)adjuvant CT type, no (%)** |  |  |
| Antracycline-Taxane based | 71(93.42) | 40(88.89) |
| Taxane -based | 5(6.58) | 5(11.11) |
| **HER2 directed therapy, no (%)** |  |  |
| Yes | 24(31.58) | 14(31.11) |
| No | 52(68.42) | 31(68.89) |
| **Hormonal therapy, no (%)** |  |  |
| Yes | 47(62.67) | 27(60.00) |
| No | 28(37.33) | 18(40.00) |
| Missing | 1 | 0 |
