# Supplementary material for: Intestinal microbiota influences clinical outcome and side effects of early breast cancer treatment
Source: Cell Death Differ. 2021 May 7;28(9):2778–96. doi: 10.1038/s41418-021-00784-1 (PMC8408230; doi:10.1038/s41418-021-00784-1)
Supplement: Supplementary file 9 — Supplementary Table 2 [file 41418_2021_784_MOESM9_ESM.docx]

| Table S2. Prognostic factors influencing axillary lymph node involvement. | | | |
| --- | --- | --- | --- |
| Groups | pN+ (n=26) | pN- (n=30) | *p* value* |
| **Age**, year, median [range] | 52.50[47.00-63.00] | 54.50[46.00-62.00] | 0.7547 |
| **Tumor size** |  |  | 0.0578 |
| pT1, no (%) | 11 (42.31) | 21 (65.63) |  |
| > pT1, no (%) | 15 (57.69) | 9 (37.50) |  |
| **SBR grade** |  |  | 0.2953 |
| Grade 1-2, no (%) | 16 (61.54) | 14 (46.67) |  |
| Grade 3, no (%) | 10 (38.46) | 16 (53.33) |  |
| **Molecular subtypes** |  |  | 0.0892 |
| RH+HER2-, no (%) | 17(65.38) | 12(40.00) | 0.0580 |
| HER2+, no (%) | 8(30.77) | 12(40.00) | 0.5796 |
| HR-/HER2-, no (%) | 1(3.85) | 6(20.00) | 0.1080 |

*Wilcoxon test for medians, all other p are CHI-2 test (Fisher test).
