# Supplementary material for: Intestinal microbiota influences clinical outcome and side effects of early breast cancer treatment
Source: Cell Death Differ. 2021 May 7;28(9):2778–96. doi: 10.1038/s41418-021-00784-1 (PMC8408230; doi:10.1038/s41418-021-00784-1)
Supplement: Supplementary file 10 — Supplementary Table 3 [file 41418_2021_784_MOESM10_ESM.docx]

| Table S3. Description of chemotherapy-related side effects declared at 12 months (any grade). | | |
| --- | --- | --- |
|  | Pre-CT^#^ samples  n=76 | Post-CT samples  n=45 |
| **Neurological*, no (%)** |  |  |
| Yes | 51(76.12) | 30(71.43) |
| No | 16(23.88) | 12(28.57) |
| Missing | 9 | 3 |
| **Gastro-intestinal, no (%)** |  |  |
| *Nausea* |  |  |
| Yes | 7(10.45) | 3(7.14) |
| No | 60(89.55) | 39(92.86) |
| Missing | 9 | 3 |
| *Diarrhea* |  |  |
| Yes | 15(22.39) | 9(21.43) |
| No | 52(77.61) | 33(78.57) |
| Missing | 9 | 3 |
| *Constipation* |  |  |
| Yes | 23(34.33) | 15(35.71) |
| No | 44(65.67) | 27(64.29) |
| Missing | 9 | 3 |
| **Rheumatological**, no (%)** |  |  |
| Yes | 37(55.22) | 23(54.76) |
| No | 30(44.78) | 19(45.24) |
| Missing | 9 |  |
| **Hot flashes, no (%)** |  | 3 |
| Yes | 42(62.69) | 21(50.00) |
| No | 25(37.31) | 21(50.00) |
| Missing | 9 | 3 |
| **Metabolic : BMI***, no (%)** |  |  |
| ≥25 | 40(62.50) | 24(61.54) |
| <25 | 24(37.50) | 15(38.46) |
| Increased BMI****_Yes | 30 (39.50) | 24 (53.33) |
| Increased BMI_No | 34 (44.73) | 15 (33.33) |
| Missing | 12 | 6 |

^#^ CT: chemotherapy; *Concentration disorder and/or dysgueusia and/or headache and/or paresthesis and/or peripheral neuropathy and/or memory disorder; ** Joint and/or muscular; ***BMI: body mass index, ****weight gain at any level.
