# Supplementary material for: Intestinal microbiota influences clinical outcome and side effects of early breast cancer treatment
Source: Cell Death Differ. 2021 May 7;28(9):2778–96. doi: 10.1038/s41418-021-00784-1 (PMC8408230; doi:10.1038/s41418-021-00784-1)
Supplement: Supplementary file 11 — Supplementary Figure and Table legends [file 41418_2021_784_MOESM11_ESM.docx]

**SUPPLEMENTAL MATERIALS**

**Supplemental Tables**

Table S1. Clinical and pathological characteristics of the CANTO sub-cohort.

Table S2. Prognostic factors influencing axillary LN involvement.

Table S3. Chemotherapy related side effects at 12 months post -adjuvant chemotherapy in CANTO sub-cohort.

**Supplemental Figures**

**Figure S1. Consort diagram of CANTO study participants.**

Consortium diagram for patient enrollment in the CANTO trial and according to microbiome analyses and type treatment (adjuvant or neo-adjuvant CT).

**Figure S2. Metagenomics analyses of stool samples from early BC patients according to the tumor size in pre- and post-adjuvant chemotherapy stool samples.**

**A-B.** Beta-diversity ordination plot based on principal coordinate analysis of normalized and standardized data of fecal microbiota composition before and after chemotherapy (pre- (**A**), n= 56 and post- (**B**) CT, n=34) in BC patients according to pathological tumor size (pT1, orange dots vs pT>1, blue dots. **C-D.** Variable importance plot (VIP) was implemented within partial least square discriminant analysis segregating patients according to pathological tumor size, describing the most discriminant species in descending order of importance in pre- (A), n=56 and post- (**B**) CT stool samples, n=34), considering bacteria with prevalence >2.5%.

The p value is indicated at the top of the Y axis. Arrows point out bacteria commonalities across graphs and clinical parameters.

**Figure S3. Metagenomics analyses of stool samples from early BC patients according to the histological grade in pre- and post-CT stool samples and in BC treated in a neoadjuvant setting.**

**A-B**. Beta-diversity ordination plot based on principal coordinate analysis of normalized and standardized data of fecal microbiota composition pre- (A), n=75 and post- (B) CT, n=44 in BC patients according to tumor histological grade SBR (Gr): Gr 1-2, orange dots vs Gr3, blue dots. The p value is indicated at the top of the Y axis. **C**. Variable importance plot (VIP) was implemented within partial least square discriminant analysis segregating patients according to tumor grade describing the most discriminant species in descending order of importance in post-CT stool samples (n=44), considering bacteria with prevalence >2.5%. **D.** Variable importance plot (VIP) was implemented within partial least square discriminant analysis, differentiating stage 0 (orange) and stage I/ II (blue) in neo-adjuvant setting, describing the most discriminant species in post-CT stool samples (n=10). Arrows point out bacteria commonalities across graphs and clinical parameters. P-values are indicated at the top of the Y axis.

**Figure S4. Metagenomics analyses of stool samples from early BC patients according to axillary lymph node involvement in pre- and post-CT stool samples.**

**A-C** Beta-diversity ordination plot based on principal coordinate analysis of normalized and standardized data of fecal microbiota composition in BC patients according to lymph node involvement: positive lymph node (pN+, blue dots) vs negative lymph node (pN-, orange dots) in stools collected before and after adjuvant CT (pre- (**A**), n=56 and post-CT (**C**), n=34). The p value is indicated at the top of the Y axis. **B-D** Variable importance plot (VIP) was implemented within partial least square discriminant analysis segregating patients according to lymph node involvement, describing the most discriminant species in descending order of importance in pre- (**B**), n=56 and post- (**D**) CT stool samples (n=34), considering bacteria with prevalence >2.5%. Arrows point out bacteria commonalities across graphs and clinical parameters. P-values are indicated at the top of the Y axis.

**Figure S5. At the cross roads between metagenomics (MG) and metabolomics (MB) pathways in plasma and stools.**

**A.** Plasma levels of kynurenine collected before and after CT (pre- and post-CT), left panel), analyzed by MB in patients with stage II-III (left panel) and stage I ( right panel) BC). **B.** Plasma levels of methionine collected before and after CT (pre- and post-CT, left panel) analyzed by MB in patients with stage II-III BC and identification of the biosynthesis of L- methionine pathway in stools collected post-CT analyzed by MG and segregated according to pathological tumor stage (pT1 vs >pT1, right panel). **C.** Plasma levels of glucose- and fructose-6 phosphate collected before and after CT (pre- and post-CT, left panel) analyzed by MB in patients with stage II-III BC and identification of the TCA pathway in stools collected post-CT, analyzed by MG and segregated according to pathological lymph node involvement (pN- vs pN+, right panel). **C.** Plasma levels of dimethylarginine collected before and after CT (pre- and post-CT, left panel) and analyzed by MB in patients with stage II-III BC and identification of the L-arginine biosynthesis pathway in post-CT stool analyzed by MG and segregated according pathological lymph node involvement (pN- vs pN+, right panel). P values analyzed by Wilcoxon test.

**Figure S6. Microbiota composition and overt weight gain assessed 12 months post-CT.**

**A-C.** Beta-diversity of the taxonomic species identified in the MG-based stool composition before and after chemotherapy (pre-CT, **A** and post-CT, **C**) in early BC patients according to the increase in weight (Yes or No) 1 year after CT. P values analyzed by Wilcoxon test. **B-D**. Variable importance plot (VIP) was implemented within partial least square discriminant analysis segregating patients according to weight gain 1 year after CT, describing the most discriminant species in descending order of importance in pre-(**B**) and post-CT (**D**) stool samples, considering bacteria with prevalence >2.5%. P-values are indicated at the top of the Y axis. Pre-CT samples (n=64) and post-CT samples (n=41).

**Figure S7. Microbiota composition and long-term side effects evaluated 12 months after BC chemotherapy.**

**A-C**. Beta-diversity in pre-CT (left panel) and post-CT (middle panel) stool samples and Shannon/richness diversity (right) in **p**ost-CT stool samples according to the presence or absence of long-term side effects of any grade evaluated with CTCAE v4 such as constipation (**A**) diarrhea (**B**), hot flashes (**C**) (refers to Table S3). P-values are indicated at the top of the Y axis.
